# L-selectin regulates human neutrophil transendothelial migration

**DOI:** 10.1242/jcs.250340

**Published:** 2021-02-08

**Authors:** Izajur Rahman, Aida Collado Sánchez, Jessica Davies, Karolina Rzeniewicz, Sarah Abukscem, Justin Joachim, Hannah L. Hoskins Green, David Killock, Maria Jesus Sanz, Guillaume Charras, Maddy Parsons, Aleksandar Ivetic

**Affiliations:** 1 BHF Centre for Research Excellence, School of Cardiovascular Medicine and Sciences, King's College London, James Black Centre, 125 Coldharbour Lane, London SE5 9NU, UK; 2 Department of Pharmacology and Faculty of Medicine and Odontology, University of Valencia, Av. Blasco Ibáñez 15, 46010 Valencia, Spain; 3 Institute of Health Research INCLIVA, University Clinic Hospital of Valencia, Av. Menéndez Pelayo 4, 46010, Valencia, Spain; 4 CIBERDEM-Spanish Biomedical Research Centre in Diabetes and Associated Metabolic Disorders, ISCIII, Av. Monforte de Lemos 3-5, 28029, Madrid, Spain; 5 London Centre for Nanotechnology, University College London, London WC1H 0AH, UK; 6 Randall Centre for Cell and Molecular Biophysics, King's College London, New Hunt's House, Guy's Campus, London SE1 1UL, UK

**Keywords:** PECAM-1, Diapedesis, Transmigration, p38 MAPK, JNK

## Abstract

The migration of circulating neutrophils towards damaged or infected tissue is absolutely critical to the inflammatory response. L-selectin is a cell adhesion molecule abundantly expressed on circulating neutrophils. For over two decades, neutrophil L-selectin has been assigned the exclusive role of supporting tethering and rolling – the initial stages of the multi-step adhesion cascade. Here, we provide direct evidence for L-selectin contributing to neutrophil transendothelial migration (TEM). We show that L-selectin co-clusters with PECAM-1 – a well-characterised cell adhesion molecule involved in regulating neutrophil TEM. This co-clustering behaviour occurs specifically during TEM, which serves to augment ectodomain shedding of L-selectin and expedite the time taken for TEM (TTT) to complete. Blocking PECAM-1 signalling (through mutation of its cytoplasmic tail), PECAM-1-dependent adhesion or L-selectin shedding, leads to a significant delay in the TTT. Finally, we show that co-clustering of L-selectin with PECAM-1 occurs specifically across TNF- but not IL-1β-activated endothelial monolayers – implying unique adhesion interactomes forming in a cytokine-specific manner. To our knowledge, this is the first report to implicate a non-canonical role for L-selectin in regulating neutrophil TEM.

## Abstract (precis)

**Highlighted Article:** Neutrophil L-selectin co-clusters with PECAM-1 in cis during transendothelial migration (TEM). Clustering neutrophil PECAM-1 activates p38 MAPK and JNK to regulate L-selectin shedding, which in turn expedites TEM.

## INTRODUCTION

The rapid recruitment of circulating neutrophils to inflamed postcapillary venules, and their subsequent transendothelial migration (TEM), is absolutely essential to the inflammatory response. The multi-step adhesion cascade embodies a paradigm that describes the increasingly adhesive steps leukocytes take with the underlying inflamed endothelium prior to their exit from the microcirculation. Recent advances in understanding how cell adhesion molecules arrange in *cis* is now building a more complex framework of molecular interactions that are triggered during the adhesion cascade. For example, in mice, P-selectin glycoprotein ligand-1 (PSGL-1, also known as SELPLG) co-clusters in *cis* with L-selectin (CD62L, also known as SELL) in lipid rafts during neutrophil rolling to subsequently activate the LFA-1 (αLβ2) integrin (Stadtmann et al., 2013) and transition slow rolling towards arrest. Although this mechanism may not be conserved in humans (Morikis et al., 2017; Pruenster et al., 2015), it nonetheless provides a plenary example of how individual cell adhesion molecules coalesce into unique ‘interactomes’ to advance the multi-step adhesion cascade.

There is excellent *in vivo* and *in vitro* evidence to suggest that 70–90% of all neutrophil TEM events adopt a paracellular route, leaving ∼10–30% of TEM events to proceed via non-junctional routes (Ley et al., 2007; Muller, 2011; Woodfin et al., 2011). During paracellular TEM, leukocytes must disrupt junctions of adjoining endothelial cells and, concomitantly, limit excessive oedema. Numerous cell adhesion molecules have been identified at adherens and tight junctions, collectively retaining barrier integrity to regulate the movement of cells and macromolecules under steady-state and inflammatory conditions. Examples include: junctional adhesion molecule (JAM)-A, -B and -C (also known as F11R or JAM1, JAM2 and JAM3, respectively); platelet-endothelial cell adhesion molecule-1 (PECAM-1, also referred to as CD31); CD99; intercellular adhesion molecule-2 (ICAM-2), vascular endothelial (VE)-cadherin, occludin, nectin and the claudins (Allport et al., 1997a; Bradfield et al., 2007; Devilard et al., 2013; Huang et al., 2006; Lou et al., 2007; Ludwig et al., 2005; Reijerkerk et al., 2006; Schenkel et al., 2006; Woodfin et al., 2009). Most adherens or tight junctions form homotypic interactions between apposing endothelial cells. Junctional disruption during TEM is facilitated in part by the presence of complementary molecules on the leukocyte surface. Therefore, cell adhesion molecules, such as PECAM-1, are subject to hetero-cellular homotypic interactions (i.e. displacing endothelial–endothelial PECAM-1 interaction with endothelial–leukocyte PECAM-1 interaction) (Privratsky et al., 2011). Studies in PECAM-1-knockout mice revealed that this molecule has a contribution to early stages of TEM (Woodfin et al., 2009).

Although signalling downstream of PECAM-1 engagement has been extensively characterised at the molecular level in both platelets and endothelial cells, little is known about signalling in human leukocytes. Using the U973 monocyte-like cell line, Florey et al. revealed that two tyrosine residues within the immunoreceptor tyrosine-based inhibition motif (ITIM) are phosphorylated when PECAM-1 is clustered during TEM to drive the transmigratory process (Florey et al., 2010). Phosphorylation of PECAM-1 at ITIM tyrosine 663 and 686 leads to its translocation into lipid rafts, where it is dephosphorylated to inactivate signal transduction during this period of TEM (Florey et al., 2010). Function-blocking antibodies to PECAM-1 (e.g. HEC7 for human and Mec13.3 for mouse PECAM-1) can impede primary neutrophil and monocyte transmigration in various *in vitro* and *in vivo* models (Christofidou-Solomidou et al., 1997; Muller et al., 1993). Interestingly, mouse knockout models of PECAM-1 have exposed conflicting data that imply strain-restricted phenotypes (Schenkel et al., 2006). The mechanistic conservation of TEM between mice and humans is therefore still unclear, in part due to the disparity in readouts amongst experimental models (i.e. complete emigration through multiple venular barriers for mouse *in vivo* assays versus entry into the subendothelial space for human *in vitro* assays).

L-selectin is expressed on most circulating leukocytes and is best known for its role in mediating tethering and rolling (Ivetic, 2018; Ivetic et al., 2019). Recently, L-selectin has been shown to exist in the protruding pseudopods of transmigrating CD14-positive human monocytes. Moreover, the pool of L-selectin within transmigrating pseudopods is clustered before it is cleaved by ectodomain shedding – suggesting the potential for L-selectin-dependent signalling during TEM (prior to shedding). L-selectin can be clustered via two distinct mechanisms – ‘outside-in’ and ‘inside-out’ – where the former is driven by classic binding to extracellular ligands and the latter is driven by intracellular signals that lead to actin remodelling and clustering of the L-selectin tails. The majority of our understanding of L-selectin clustering is derived from studies using monoclonal antibodies or extracellular ligands to drive outside-in clustering (reviewed extensively in Ivetic et al., 2019). In this study, we asked whether inside-out modes of L-selectin clustering could be influenced by other cell adhesion molecules involved in the adhesion cascade. Indeed, we discovered that clustering of PECAM-1 promoted the subsequent clustering of L-selectin. Moreover, these receptors co-cluster in *cis* in both primary human monocytes and neutrophils. The relationship of co-clustering between PECAM-1 and L-selectin was found to be unidirectional and non-reciprocal – further suggesting that co-clustering of these molecules is context specific (e.g. during TEM, when PECAM-1 is engaged and clustered, rather than rolling when L-selectin is engaged and transiently clustered). Fluorescence lifetime imaging microscopy (FLIM) revealed that L-selectin–RFP and PECAM-1–GFP co-clustered within distances of less than 10 nm in differentiated HL-60 (dHL-60) cells actively engaged in TEM under flow conditions. Moreover, L-selectin–PECAM-1 co-clustering was only witnessed in cells crossing TNF-, but not IL-1β-, activated endothelial monolayers. Direct antibody-mediated clustering of PECAM-1 on neutrophils potentiated ectodomain shedding of L-selectin. Moreover, the Akt family kinases, JNK family kinases and p38 MAPKδ (also known as MAPK13) were robustly activated following clustering of neutrophil-derived PECAM-1 – suggesting a contribution of these downstream effectors in regulating L-selectin shedding during TEM. Blocking L-selectin shedding with the small-molecule inhibitor TAPI-0, resulted in significantly slower neutrophil transmigration times across TNF- but not IL-1-β-activated endothelial monolayers, implying that L-selectin shedding optimises neutrophil TEM in a cytokine-specific manner. Taken together, we suggest that neutrophil TEM across TNF-activated endothelial monolayers drives the co-clustering of PECAM-1 and L-selectin to potentiate its shedding and expedite the time taken for TEM (TTT). To our knowledge, this study provides the first evidence for the direct involvement of L-selectin in regulating neutrophil TEM.

## RESULTS

### PECAM-1 clustering drives inside-out clustering of L-selectin

**Fig. 1.**
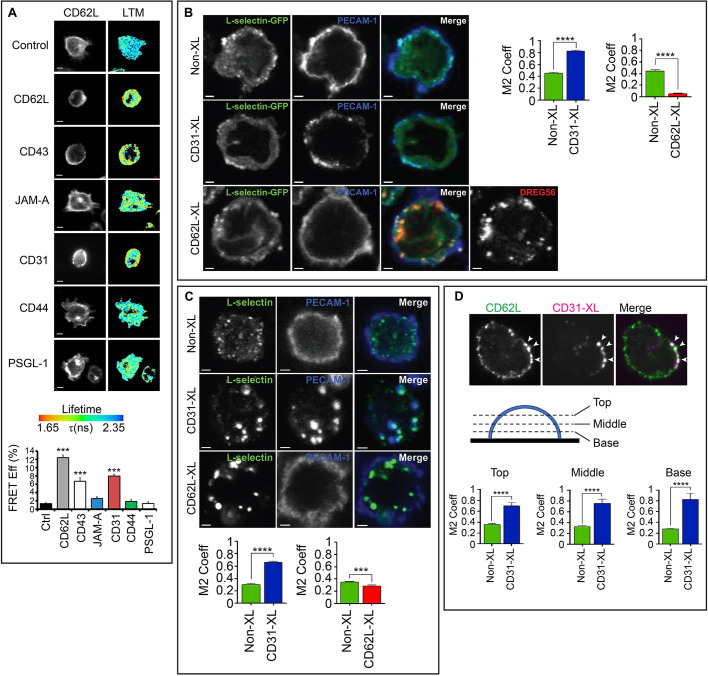
**Antibody-mediated clustering of PECAM-1 drives inside-out clustering of L-selectin and its co-clustering with PECAM-1.** (A) THP-1 cells expressing L-selectin–GFP and L-selectin–RFP were treated with a range of primary antibodies targeting L-selectin (CD62L), CD43, JAM-A, PECAM-1 (CD31), CD44 or PSGL-1. Primary antibodies were subsequently clustered with secondary antibody and cells plated onto poly-L-lysine (PLL), fixed in 4% PFA and prepared for FRET/FLIM analysis. GFP fluorescence channel and lifetime (LTM) images are provided for each cell line. The lifetime of fluorescence is expressed in a pseudocolour scale from red (low lifetime with a very high probability of interaction) to blue (high lifetime with a very low probability of interaction). Bar graph represents mean±s.e.m. acquired from at least 45 cells across three independent experiments. ***P<0.001 (one-way ANOVA followed by Tukey's post-test). Scale bars: 5 μm. (B) Confocal microscopy was used to monitor the effect of clustering either PECAM-1 (CD31-XL) or L-selectin–GFP (CD62L) on THP-1 cells. At least 200 cells were analysed. Manders’ overlap coefficient (M2 Coeff) was used to quantify (mean±s.e.m.) the extent of overlap between signals corresponding to CD31 and CD62L, represented in the column scatter graph. ****P<0.0001 (two-tailed unpaired Student's *t*-test with Welch's correction). Scale bars: 2.5 μm. (C) Primary human CD14-positive monocytes were purified from healthy donor bloods and clustering of either CD31 or CD62L were performed as described in B. M2 Coeff was used as a measure of colocalization (mean±s.e.m.) between signals corresponding to CD31 and CD62L, represented in the column scatter graph. At least 200 cells were analysed per treatment across at least three independent experiments. ***P<0.001, ****P<0.0001 (two-tailed unpaired Student's *t*-test with Welch's correction used). Scale bars: 2.5 μm. (D) iSIM was used to monitor the impact of clustering CD31 on the co-clustering behaviour of CD62L in primary human neutrophils. Cells were left untreated (Non-XL) or antibodies used to cluster CD31 (CD31-XL) as in B. Cells were plated onto poly-L-lysine and incubated at 37°C before fixation in 4% PFA. LAM1-14 monoclonal antibody was used to stain endogenous L-selectin, followed by donkey anti-mouse-IgG secondary antibody conjugated to Alexa Fluor^®^ 488. A representative iSIM cross-sectional view of a neutrophil clustered with primary anti-CD31 antibody and secondary donkey anti-sheep antibody conjugated to Alexa Fluor^®^ 633 is shown. Arrowheads highlight regions of L-selectin that are co-clustering with cross-linked PECAM-1. Three different optical sections were taken, as depicted in the diagram, and Manders' overlap co-efficient (M2 Coeff) was used to quantify the extent of overlap between the two signals. Mean±s.e.m. are derived from 38 cells for each treatment across three independent experiments. ****P<0.0001 (two-tailed unpaired Student's *t*-test with Welch's correction). Images shown are 13.2×13.2 μm (E) Flow cytometric analysis of primary human neutrophils treated with the anti-PECAM-1 monoclonal antibody HEC7. Neutrophils were treated with HEC7 alone (grey line) or HEC7 plus a secondary clustering antibody conjugated to Alexa Fluor^®^ 647 (green line). DREG56 directly conjugated to phycoerythrin (PE) was used to monitor L-selectin expression in untreated cells (black line), and cells with HEC7 alone (grey line) or with HEC7 plus clustering secondary antibody (green line). Purple line represents unstained neutrophils. Histogram is representative of three independent experiments (see Figs S1 and S2).

To understand whether non-integrin cell adhesion molecules contribute to inside-out clustering of L-selectin, THP-1 cells were engineered to co-express two forms of wild-type (WT) L-selectin: C-terminally tagged with either GFP or RFP. Primary antibodies targeting specific cell adhesion molecules (CD43, JAM-A, PECAM-1, CD44 and PSGL-1) were used, in combination with secondary antibody, to drive their clustering (see Materials and Methods for details). Using FLIM, we could quantify increases in FRET efficiency between the GFP and RFP tags that, in turn, reflect L-selectin clustering. Antibody-mediated clustering of L-selectin with the monoclonal antibody DREG56, promoted maximal FRET efficiency in THP-1 cells co-expressing L-selectin–GFP and L-selectin–RFP to ∼12% (Fig. 1A). In THP-1 cells, clustering of endogenous PSGL-1 had no influence on the clustering of L-selectin–GFP and L-selectin–RFP, which is in agreement with a recent report by Morikis et al. (2017). We however noticed that antibody-mediated clustering of either CD43 or PECAM-1 significantly increased the FRET efficiency of the L-selectin–GFP and L-selectin–RFP tags in THP-1 cells to 7% and 8%, respectively (Fig. 1A). Given that monocyte and neutrophil PECAM-1 have a well-established role in TEM (Muller et al., 1993; Thompson et al., 2001), and to determine whether L-selectin plays a direct role in regulating TEM, we focused exclusively on the relationship between L-selectin and PECAM-1. Moreover, studying the contribution of PECAM-1 to driving the co-clustering of L-selectin aligned with our recent interests in understanding how L-selectin contributes to monocyte protrusive behaviour in TEM (Newe et al., 2019; Rey-Gallardo et al., 2018; Rzeniewicz et al., 2015). To further determine whether antibody-mediated clustering of PECAM-1 caused L-selectin to cluster locally or distally to PECAM-1 clusters, laser scanning confocal microscopy was used to quantify the extent of overlap in fluorescence signals corresponding to PECAM-1 and L-selectin. Manders’ overlap coefficiency (M2 Coeff) revealed that the extent of co-clustering between L-selectin and PECAM-1 increased significantly following antibody-mediated clustering of PECAM-1 (Fig. 1B). Reciprocal experiments involving antibody-mediated clustering of L-selectin did not lead to the corresponding co-clustering of PECAM-1 (Fig. 1B), implying a unidirectional relationship in co-clustering behaviour. The co-clustering of L-selectin with clusters of PECAM-1 could be recapitulated in both primary human monocytes (Fig. 1C) and neutrophils (Fig. 1D; Movie 1). Whereas the majority of L-selectin–PECAM-1 clusters were present on the plasma membrane of primary neutrophils, a small amount localised to intracellular compartments (such as vesicles), suggestive of receptor internalisation (Movie 1). Quantification by means of flow cytometry of neutrophil L-selectin confirmed that surface L-selectin expression decreased by 3.85% when just anti-PECAM-1 antibody was used, which increased to 22% upon crosslinking with secondary antibody (Fig. 1E; Figs S1, S2).

### Co-clustering of L-selectin with PECAM-1 during TEM is cytokine specific

Many *in vitro* transmigration assays have employed HUVEC monolayers stimulated with either TNF or IL-1β, with the broad assumption that the molecular mechanism guiding human neutrophil TEM is similar. Based on previous work in mice, where a cytokine-specific role for PECAM-1-dependent migration through venular walls has been documented (Thompson et al., 2001), we were motivated to explore whether the cytokines TNF or IL-1β could play divergent roles in driving the co-clustering of L-selectin with PECAM-1 – specifically during TEM. We have successfully used THP-1 cells to investigate mechanisms regulating L-selectin clustering in TEM, and its interaction with calmodulin or ERM proteins during TEM (Newe et al., 2019; Rey-Gallardo et al., 2018; Rzeniewicz et al., 2015). Owing to their size, the majority of THP-1 cells are trapped in mid-TEM and rarely complete TEM (i.e. <1% of all THP-1 cells undergo full TEM). While this cellular model captures a high degree of cells in mid-TEM, so that the spatio-temporal organisation of interaction between L-selectin and binding partners can be closely interrogated, it precludes quantification of TEM to completion. In contrast, the neutrophil-like HL-60 cell line is smaller in size than THP-1 cells. However, they rarely transmigrate across activated endothelial monolayers. We noticed that stable expression of the chemokine receptor, CXCR2, dramatically improves TEM efficiency to ∼80% (see Fig. S2). This observation has been recently reported by others (Yadav et al., 2018) and clearly reveals the essential role of CXCR2 in driving TEM of HL-60 cells. Both L-selectin and PECAM-1 are poorly expressed in HL-60 cells, which allowed us to develop a cellular model to interrogate their respective contribution to TEM (see Fig. S3 and S4). Using this cell line, we could test if the stable expression of L-selectin-RFP and PECAM-1–GFP would drive their co-clustering specifically during TEM.

**Fig. 2.**
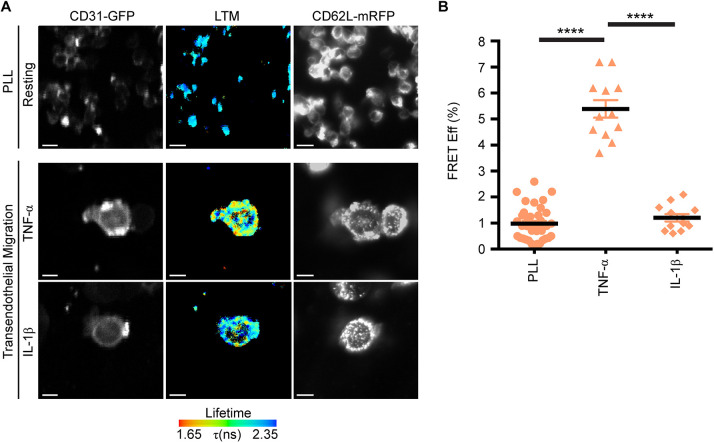
**Co-clustering of L-selectin with PECAM-1 occurs exclusively during TEM in a cytokine-specific manner.** (A) dHL-60 cells co-expressing L-selectin–RFP and PECAM-1–GFP were plated onto poly-L-lysine (PLL) at 37°C for 5 min and then rapidly fixed in 4% PFA. FLIM was used to monitor the FRET efficiency between the GFP and RFP tags. (B) Differentiated HL-60 cells co-expressing PECAM-1–GFP and L-selectin–RFP were perfused over TNF- or IL-1β-activated HUVECs. Cells were rapidly fixed in 4% PFA when the majority of cells were observed undergoing mid-TEM. Given that endothelial PECAM-1 is enriched at cell-to-cell junctions, FLIM/FRET measurements were acquired in dHL-60 cells captured in mid-TEM (i.e. within regions that were neither exclusively above nor below the endothelium). Panels display the green channel for PECAM-1–GFP (left column), the GFP lifetime (middle column, LTM) and the red channel for L-selectin–RFP (right column). The fluorescence lifetime of GFP is provided as a pseudocolour scale of blue (long lifetime=little to no FRET) to red (short lifetime=FRET). Quantification (mean±s.e.m.) of FRET efficiency between PECAM-1–GFP and L-selectin–RFP was acquired through an average measurement from 35 cells analysed under resting conditions and from 12 cells caught in mid-transmigration across either TNF- or IL1-β-activated HUVECs. Measurements are derived from three independent experiments. ****P<0.0001 (one-way ANOVA followed by multiple comparisons corrected for by the Sidak method). Scale bars: 5 μm.

HL-60 cells stably expressing L-selectin–RFP and PECAM-1–GFP were seeded onto poly-L-lysine (PLL)-coated coverslips to monitor the extent of FRET between the GFP and RFP tags using FLIM. Negligible co-clustering between PECAM-1–GFP and L-selectin–RFP was witnessed within a sub-10 nm resolution (0.982±0.111%, mean±s.e.m., Fig. 2A, upper row). This cell line was subsequently differentiated using 1.3% DMSO (hereon termed dHL-60 cells; see Materials and Methods for further details) and perfused over activated endothelial monolayers to assess co-clustering by means of FRET. Indeed, a 5-fold increase in FRET efficiency between the GFP and RFP tags (5.392±0.338%) was observed when cells were perfused over TNF-activated HUVECs (Fig. 2A, middle row and Fig. 2B). In contrast, perfusion of the same stable cell line over IL-1β-activated HUVECs led to negligible changes in FRET efficiency compared to cells adhering to PLL (1.208±0.142%; Fig. 2A, lower row and Fig. 2B).

### L-selectin shedding is triggered in transmigrating neutrophils in a cytokine-specific manner

**Fig. 3.**
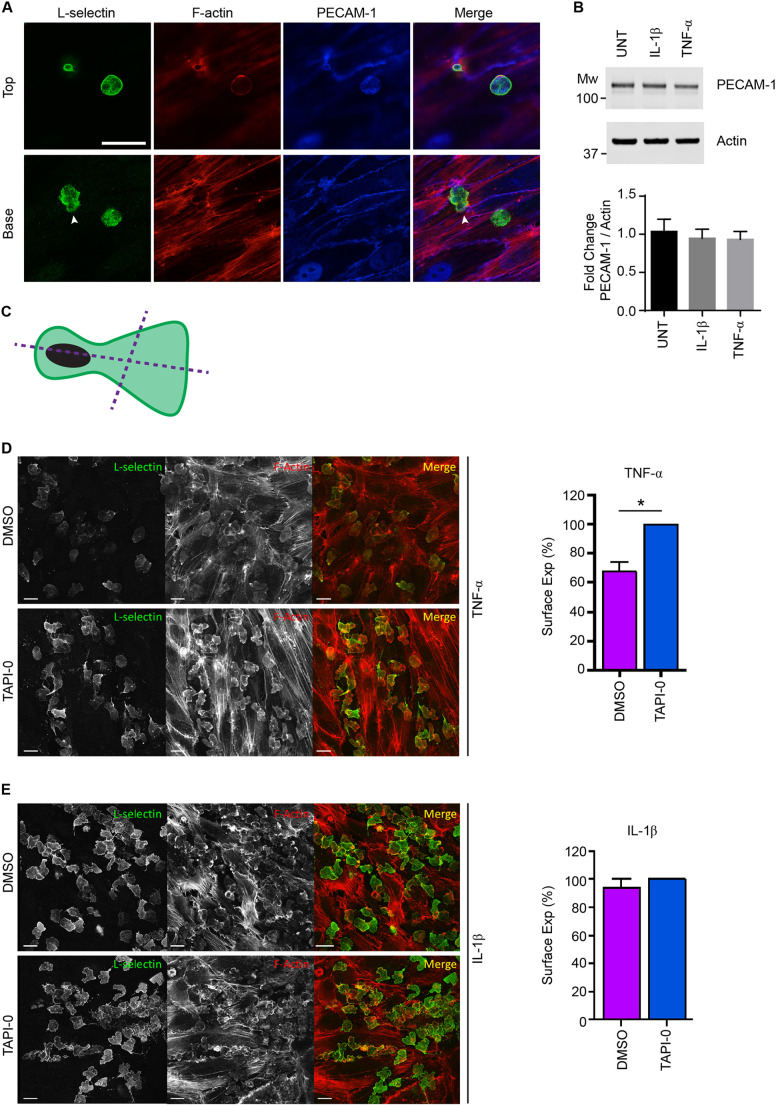
**L-selectin shedding is activated in neutrophils crossing TNF-, but not IL-1β-, activated HUVECs.** (A) Primary human neutrophils were perfused over TNF-activated HUVEC monolayers and after 6 min of perfusion were fixed in 4% PFA and prepared for immunostaining for PECAM-1 (blue, Alexa Fluor^®^ 633), L-selectin (green, Alexa Fluor^®^ 488) and F-actin (red, TRITC–phalloidin). Two primary human neutrophils are captured in different stages of the multi-step adhesion cascade – firm adhesion (right-hand side) and mid-TEM (left-hand side) under flow conditions. Two optical sections, Top and Base, reveal the subcellular organisation of endogenous L-selectin present within the transmigrated pseudopod (arrowhead) and the non-transmigrated cell body (or uropod). Distinct morphological characteristics of flat pseudopodial extensions in the subendothelial space and rounded non-transmigrated body afford easy interpretation of transmigrated and non-transmigrated regions. Anti-PECAM-1 staining of TNF-activated HUVEC reveals the well-documented discontinuity of the signal specifically at the region of paracellular TEM. Note that L-selectin expression is present in both the transmigrated pseudopod and non-transmigrated uropod, which has been observed previously in primary human (CD14-positive) monocytes (Rzeniewicz et al., 2015). Scale bar: 15 μm. (B) Analysis of HUVEC PECAM-1 protein expression through western blot analysis, following overnight stimulation with or without the cytokines IL-1β and TNF (UNT, untreated). An anti-actin western blot is used as a loading control for PECAM-1. Bar graph represents the mean±s.d. band intensity values corresponding to PECAM-1, after normalisation against the actin western blot bands (*n*=3 independent experiments). (C) Schematic representation of how line intensity profiles were drawn and quantified for neutrophils within the subendothelial space. More details are provided in the Materials and Methods section. (D) Primary human neutrophils, pre-treated for 20 min with either DMSO or 10 µM TAPI-0, were perfused over TNF-activated HUVEC monolayers at a density of 10^6^ cells per ml. Cells were perfused for 5 min, followed by a further 15 min with neat HL-60 medium. By the end of perfusion, over 95% of all neutrophils had successfully completed TEM (see Movie 2). Specimens were immediately fixed in 4% PFA prior to permeabilisation and immunostaining for L-selectin with LAM1-14 and TRITC-phalloidin. Bar graph (mean±s.e.m.) showing L-selectin surface expression, quantified as a percentage of the LAM1-14 signal that is relative to the maximal L-selectin signal assigned to the fluorescence intensity in the TAPI-0 treatment group (set at 100%). At least 420 cells were analysed per group across three independent experiments. *P<0.05 (two-tailed unpaired Student's *t*-test with Welch's correction). Scale bars: 10 μm. Images shown are (E) Primary human neutrophils were perfused over IL-1β-activated HUVEC using the same method as in D. Fluorescence intensity of the LAM1-14 signal corresponding to L-selectin was assessed through analysing line intensity profiles using Volocity software (see C and Materials and Methods). Bar graph (mean±s.e.m.) showing L-selectin surface expression, quantified as a percentage of the LAM1-14 signal that is relative to the maximal L-selectin signal assigned to the fluorescence intensity in the TAPI-0 treatment group (set at 100%). At least 420 cells were analysed per group across three independent experiments. No significant difference was seen (two-tailed unpaired Student's *t*-test with Welch's correction). Scale bars: 10 μm.

The TEM of neutrophils across activated HUVECs is known to drive ectodomain shedding of L-selectin via a disintegrin and metalloproteinase (ADAM)17 sheddase activity (Allport et al., 1997b; Smith et al., 1991). Flow cytometric analysis of L-selectin on fully transmigrated neutrophils, retrieved after subjection to static TEM assays (i.e. in the absence of shear stress), revealed that they are predominantly negative for L-selectin (Allport et al., 1997b). To monitor the extent of L-selectin shedding from primary human neutrophils transmigrating into the subendothelial space of TNF- or IL1-β-activated HUVEC monolayers, cells were perfused for 20 min and immediately fixed in 4% paraformaldehyde (PFA). Specimens were subsequently permeabilised and stained for L-selectin using the LAM1-14 monoclonal antibody (Spertini et al., 1991), followed by detection with fluorescently conjugated secondary antibody. Neutrophils captured in mid-TEM retained expression of L-selectin in both pseudopods and uropods (Fig. 3A) – consistent with what has been documented in CD14^+^ human monocytes (Rzeniewicz et al., 2015). Of note, PECAM-1 protein expression did not change between untreated (UNT) or cytokine-activated endothelial monolayers (Fig. 3B). To better interrogate the extent of L-selectin shedding in fully transmigrated neutrophils, similar experiments were conducted with 10 µM TNF protease inhibitor (TAPI-0) supplemented into the perfusate (see Materials and Methods for more detail). Direct comparisons in the percentage change of LAM1-14 fluorescence intensities were quantified between neutrophils crossing TNF- or IL1-β-activated HUVECs – with and without TAPI-0 (see Materials and Methods for more detail and Fig. 3C). Neutrophils within the subendothelial space of TNF-activated HUVEC monolayers retained 67.53±6.5% (mean±s.e.m.) of the LAM1-14 fluorescence signal compared to TAPI-0-treated neutrophils (Fig. 3D). These observations imply ectodomain shedding is triggered, but not as robustly as previously reported from other indirect methods (e.g. Allport et al., 1997b). In contrast, no significant reduction in LAM1-14 fluorescence was observed in neutrophils crossing IL-1β-activated HUVECs (Fig. 3E). Taken together, these data demonstrate cytokine-specific effects on L-selectin shedding in neutrophils that have entered the subendothelial space. These results further show that L-selectin is turned over differently between human neutrophils and CD14^+^ monocytes, where the extent of L-selectin shedding in monocytes appears to be much higher (Rzeniewicz et al., 2015).

### Antibody-mediated clustering of PECAM-1 sensitises L-selectin shedding in primary human neutrophils

**Fig. 4.**
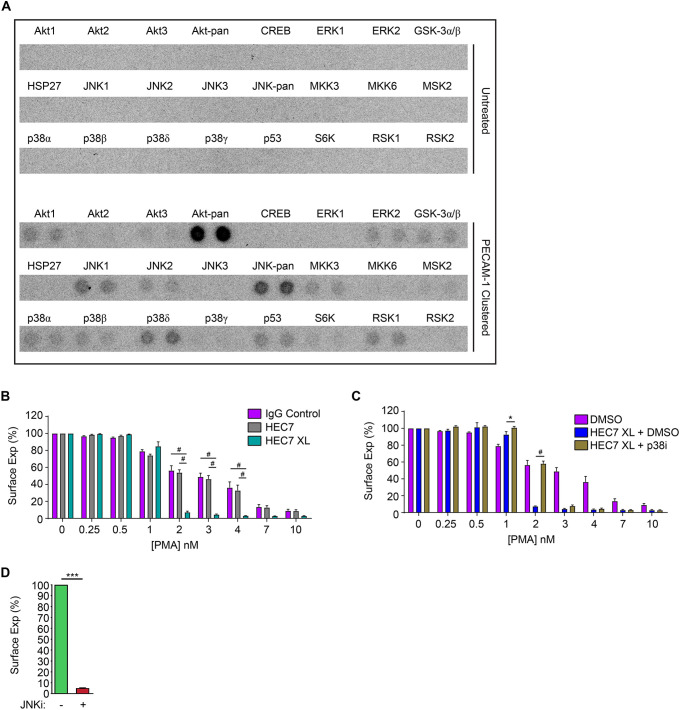
**Clustering neutrophil PECAM-1 activates Akt** **family kinases and** **MAPKs, and potentiates ectodomain shedding of L-selectin.** (A) MAPK array (R&D Systems) of primary human neutrophils clustered with HEC7 and subsequently cross-linked with secondary antibody. Upper panel represents untreated cells and lower panel is PECAM-1-clustered cells. See Materials and Methods section for more detail. (B) Control neutrophils isolated from healthy volunteers were labelled with either control IgG (purple bars), HEC7 (grey bars) or HEC7 plus clustering secondary antibody (turquoise bars). All labelling procedures were performed in the presence of Fc receptor block to avoid any non-specific binding and clustering of neutrophil Fc receptors (see Materials and Methods for more details). Antibody-labelled neutrophils were subsequently challenged with an increasing range of PMA concentrations (0.25–10 nM) and incubated for 30 min at 37°C. Alexa Fluor^®^ 647-conjugated DREG56 monoclonal antibody was used to monitor L-selectin expression by flow cytometry. Fluorescence values were compared against untreated cells, which in turn were normalised to a value of 100%. Bar graph shows mean±s.e.m. of three independent experiments using three different donors. ^#^*P*<0.0001 (two-way ANOVA followed by multiple comparisons corrected for by the Sidak method). (C) Neutrophils isolated from healthy donors were subjected to IgG control (purple bars) and HEC7 labelling as in B. Prior to and during the secondary antibody labelling procedure, which crosslinks PECAM-1 on neutrophils, cells were concomitantly incubated with either DMSO (carrier control) (blue bars) or 25 µM of the p38 MAPK inhibitor SB202190 (p38i, gold bars) at 37°C. Cells were then subjected, as in B, to increasing doses of PMA at 30°C for 30 min and Alexa Fluor^®^ 647-conjugated DREG56 monoclonal antibody was used to monitor L-selectin expression by flow cytometry. Fluorescence values were compared against untreated cells, which in turn were normalised to 100%. Bar graph shows mean±s.e.m. of 10,000 events, each collected over three independent experiments using three different donors. *P<0.05; ^#^*P*<0.0001 (two-way ANOVA followed by multiple comparisons corrected for by the Sidak method). (D) Neutrophils were incubated with either DMSO carrier (green bar) or 50 µM of the JNK inhibitor SP600125 (JNKi, red bar) for 30 min at 37°C. L-selectin levels were subsequently quantified by flow cytometry. Data represents the mean±s.e.m. of at least three independent experiments and all data were collected in triplicate. ***P<0.001 (unpaired Student's *t*-test).

We and others have previously shown that L-selectin shedding can be activated by the PKC or p38 MAPK signalling pathways (Killock and Ivetic, 2010). Using a MAPK array, we could show that clustering of PECAM-1 could activate p38 MAPKδ and JNK family proteins (Fig. 4A), suggesting that these kinases may have an influence in regulating L-selectin shedding. Phospho-Akt (Akt1–Akt3) was also strongly detected in response to PECAM-1 clustering, which is in keeping with a previous finding that PECAM-1 can co-precipitate the p85 subunit (PIK3R1) of phosphoinositide 3-kinase (PI3K) (Pellegatta et al., 1998). To further ascertain whether PECAM-1 clustering during TEM could augment ectodomain shedding of L-selectin, we clustered PECAM-1 with the monoclonal antibody HEC7 on freshly isolated neutrophils (to mimic homotypic ligation via Ig domains 1 and 2) – with and without further clustering with secondary monoclonal antibody. Cells were subsequently challenged with increasing concentrations of phorbol-12′-myristate-13′-acetate (PMA; a potent inducer of L-selectin shedding) to activate PKC. Neutrophils pretreated with HEC7 antibody did not influence PMA-induced shedding any differently to neutrophils labelled with control IgG (Fig. 4B). In contrast, clustering HEC7 with secondary antibody dramatically sensitised PMA-induced shedding of L-selectin on neutrophils (Fig. 4B). We next asked whether p38 MAPKs played a role in sensitising L-selectin shedding following clustering of PECAM-1. Preincubation of neutrophils with 25 µM of the p38 MAPK inhibitor SB202190 reverted the L-selectin shedding profile to neutrophils treated with either control antibody or HEC7 antibody (Fig. 4C). Interestingly, inhibition by SB202190 was only observed at 2 nM PMA. This result would suggest that increasing the concentration of PMA would override the inhibitory effect of SB202190. Taken together, our data suggest that L-selectin shedding is sensitised by PECAM-1 clustering and this mechanism likely involves p38 MAPKδ. Our attempts to assess blocking JNK activity in similar assays showed that the JNK inhibitor (JNKi) SP600125 was sufficient to promote L-selectin shedding without any prior clustering of PECAM-1 (Fig. 4D). These data imply that both JNK and p38 MAPK signalling may act in opposition to regulate ectodomain shedding when PECAM-1 is clustered during TEM (see Discussion section).

### Co-expression of L-selectin and PECAM-1 expedites TTT across TNF- but not IL-1β-activated HUVEC monolayers

**Fig. 5.**
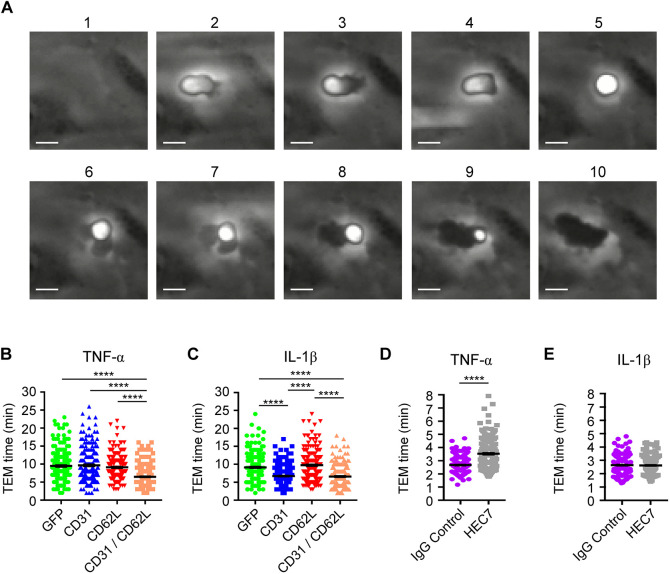
**Co-expression of L-selectin and PECAM-1 in dHL-60 cells significantly increases TTT across TNF-activated HUVECs, and functionally blocking PECAM-1 in primary human neutrophils significantly reduces TTT across TNF-activated HUVEC.** (A) Time series of frames (from Movie 3, which includes the time code) showing how TEM of HL-60 cells is quantified. Cells transitioning from phase bright (image 2) to completely phase dark (image 10). Scale bars: 5 µm. (B) Quantification of average TTT for dHL-60 cells transmigrating across TNF-activated HUVECs. The dHL-60 cells analysed are stably expressing GFP alone (green column scatter), PECAM-1–GFP alone (blue column scatter), L-selectin–RFP alone (red column scatter) or fluorescently tagged constructs of both PECAM-1 and L-selectin (orange column scatter). The TTT of at least 180 cells were quantified (mean±s.e.m.) per group across three independent experiments. ****P<0.0001 (one-way ANOVA followed by multiple comparisons corrected for by the Sidak method). (C) Quantification of mean±s.e.m. TTT for dHL-60 cells transmigrating across IL-1β-activated HUVEC. The dHL-60 cells analysed are stably expressing GFP alone (green column scatter), PECAM-1–GFP alone (blue column scatter), L-selectin–RFP alone (red column scatter) or fluorescently tagged constructs of both PECAM-1 and L-selectin (orange column scatter). The TTT of at least 180 cells were quantified per group across three independent experiments. ****P<0.0001 (one-way ANOVA followed by multiple comparisons corrected for by the Sidak method). (D) Primary human neutrophils were pre-treated with either 1 µg/ml anti-PECAM-1 function-blocking antibody (HEC7) or isotype-matched control IgG2a (IgG Control) at 37°C for at least 20 min prior to perfusion over TNF-activated HUVECs. TTT were recorded using wide-field time-lapse microscopy. Column scatter graph represents the mean±s.e.m. TTT of 140 and 145 cells for IgG2a and HEC7 treatment groups, respectively. Data is collected over three independent experiments using three different donors. ****P<0.0001 (two-tailed unpaired Student's *t*-test with Welch's correction). (E) Primary human neutrophils were pre-treated with either 1 µg/ml anti-PECAM-1 function-blocking antibody (HEC7) or isotype-matched control IgG2a (IgG Control) at 37°C for at least 20 min prior to perfusion over IL-1β-activated HUVECs. TTT were recorded using wide-field time-lapse microscopy. Column scatter graph represents the mean±s.e.m. TTT of 140 and 145 cells for IgG2a and HEC7 treatment groups, respectively. Data is collected over three independent experiments using three different donors.

As L-selectin–PECAM-1 co-clustering occurred exclusively during TEM (Fig. 2), we next sought to address whether this co-clustering impacted TTT. Compared to THP-1 cells, HL-60 cells express negligible PECAM-1 and L-selectin (see Fig. S3). Stable dHL-60 cell lines expressing GFP alone (control cell line), or single and double combinations of PECAM-1–GFP and L-selectin–RFP, were perfused over TNF- or IL-1β-activated HUVECs, and the time taken from initial capture to complete TEM was recorded and quantified (see Fig. 5A and Movie 3 for visual representation of ‘phase bright’ captured cells and ‘phase dark’ fully transmigrated cells). dHL-60 cells expressing GFP alone (which acted as control cells for lentivirally transduced dHL-60 cells) possessed similar TEM times across either TNF- or IL-1β-activated HUVEC monolayers [i.e. 9 min 29 s (±20 s, s.e.m.) for TNF versus 9 min 9 s (±18 s) for IL-1β]. These TEM times were similar to those for dHL-60 cells expressing a single cell adhesion molecule and crossing TNF-activated HUVEC monolayers [L-selectin–RFP=9 min 5 s (±16 s); PECAM-1–GFP=9 min 40 s (±22 s)]. In contrast, dHL-60 cells co-expressing L-selectin–RFP and PECAM-1–GFP displayed significantly reduced TTT – taking 6 min 28 s (±14 s) to cross TNF-activated HUVEC monolayers (Fig. 5B). Interestingly, dHL-60 cells expressing just PECAM-1–GFP undertook TTT across IL-1β-activated HUVEC monolayers in 6 min 43 s (±13 s) – yet took 9 min 40 s (±22 s) to cross TNF-activated monolayers (Fig. 5C). These data strongly suggest that stable expression of PECAM-1–GFP is sufficient to drive the faster TTT phenotype across IL-1β-activated HUVEC monolayers (Fig. 5C).

To further understand the importance of this mechanism in primary human neutrophils, we used HEC7 to selectively block neutrophil PECAM-1 and determine whether TEM was impacted in a cytokine-specific manner. HEC7 targets the first two N-terminal immunoglobulin (Ig) domains in PECAM-1, which are responsible for supporting homophilic interaction and clustering *in trans* (Paddock et al., 2016) (i.e. between adjoining endothelial cells). In agreement with the dHL-60 cellular model, HEC7 retarded the TTT for primary human neutrophils crossing TNF- but not IL-1β-stimulated HUVEC monolayers [from 2.6 min (±3 s) to 3.5 min (±6 s); Fig. 5D,E]. These data suggest the Ig domains 1 and 2 of neutrophil PECAM-1 are important to expedite human neutrophil TEM across TNF- but not IL-1β-activated HUVEC monolayers.

### Blocking ectodomain shedding of L-selectin reduces TTT across TNF-, but not IL-1β-, activated HUVECs

**Fig. 6.**
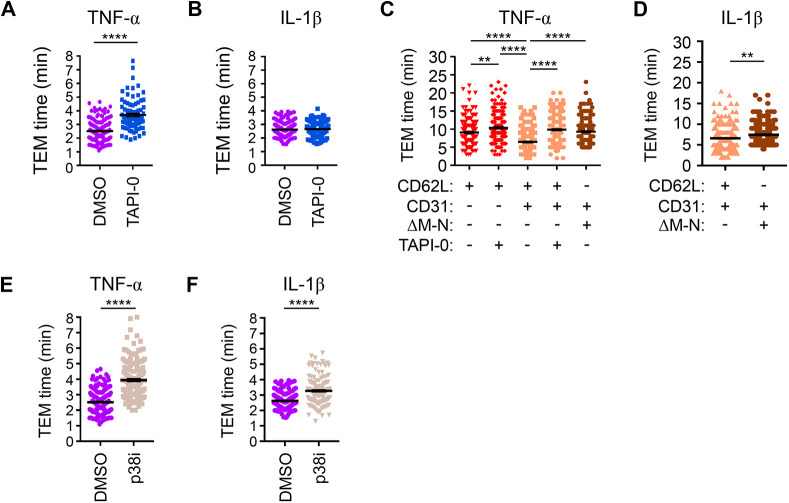
**Blocking L-selectin shedding in neutrophils and dHL-60 cells significantly delays TTT across TNF-, but not IL-1β-, activated HUVEC monolayers.** Primary human neutrophils were pre-treated either with carrier (DMSO) or 10 μM TAPI-0 at 37°C for 20 min prior to perfusion over (A) TNF- or (B) IL-1-β-activated HUVECs. Note that in all experiments either carrier or inhibitor was supplemented into the perfusate. The mean±s.e.m. TTT of neutrophils crossing TNF-activated endothelial monolayers was derived from 150 and 90 cells for DMSO and TAPI-0 treatment, respectively. The mean±s.e.m. time taken for IL1-β-dependent TEM was derived from 150 cells crossing either DMSO or TAPI-0-treatment groups. All measurements were derived from three independent experiments. ****P<0.0001 (two-tailed unpaired Student's *t*-test with Welch's correction). (C) dHL-60 cells stably expressing L-selectin–RFP alone or with PECAM-1–GFP were perfused over TNF-activated HUVECs in the absence or presence of 10 μM TAPI-0. The TTT of HL-60 cells were quantified as in Fig. 5B. Column scatter graph in C is the mean±s.e.m. measurement from 180 cells per group and derived from three independent experiments. **P<0.01; ****P<0.0001 (one-way ANOVA followed by multiple comparisons corrected for by the Sidak method). (D) dHL-60 cells stably expressing PECAM-1–GFP with either WT L-selectin–RFP or ΔM-*N* L-selectin–RFP were perfused under conditions described in Fig. 5C and specifically over IL1-β-activated HUVEC. A mean±s.e.m. measurement from 180 cells was obtained per group and derived from three independent experiments. **P<0.01 (two-tailed unpaired Student's *t*-test with Welch's correction). (E,F) Primary human neutrophils were preincubated with 25 mM of the p38 MAPK inhibitor SB202190 at 37°C for 20 min prior to perfusion over either TNF- or IL-1-β-activated HUVECs. Note that the same concentration of the inhibitor was supplemented into the perfusate during the flow assay. Mean±s.e.m. TTT swere calculated for neutrophils crossing TNF- (E) or IL-1-β- (F) activated HUVECs and compared with DMSO control groups from A and B. ****P<0.0001 (two-tailed unpaired Student's *t*-test with Welch’s correction).

To further explore the relationship between L-selectin co-clustering with PECAM-1 during TEM, L-selectin shedding and the TTT, we closely examined the time it took for primary human neutrophils to fully complete TEM across TNF-activated HUVEC monolayers in the presence or absence of TAPI-0. In the presence of carrier alone (DMSO), neutrophils would average TTT of 2.5 min (±3 s), which would increase significantly to 3.7 min (±7 s) in the presence of TAPI-0 (Fig. 6A). In contrast, perfusion of neutrophils over IL-1β-activated HUVEC monolayers led to averaged TTT of 2.6 min (±2 s) in the presence of DMSO carrier and 2.7 min (±2 s) in the presence of TAPI-0 (Fig. 6B). These data strongly imply that L-selectin shedding is triggered during TEM across TNF-activated HUVECs to expedite TTT, which is not the case for neutrophils crossing IL-1β-activated HUVEC monolayers.

We next addressed whether the expression of a non-cleavable mutant of human L-selectin, called ΔM-N L-selectin (Chen et al., 1995), could phenocopy the reduction in TTT characterised in neutrophils (Fig. 6A,B; see Materials and Methods for information on ΔM-N L-selectin). Perfusion of dHL-60 cells, stably expressing just WT L-selectin–RFP, over TNF-activated HUVEC possessed an average TTT of 9 min 5 s (±16 s). This time was further delayed to 10 min 21 s (±18 s) when TAPI-0 was supplemented into the perfusate (Fig. 6C). Perfusion of dHL60 cells co-expressing L-selectin–RFP and PECAM-1–GFP, as shown before (Fig. 5B), had TTT of 6 min 28 s (±14 s), which reduced significantly to 9 min 51 s (±16 s) when TAPI-0 was supplemented into the perfusate (Fig. 6C). Taken together, these results phenocopied the effect of using TAPI-0 on neutrophils crossing TNF-activated HUVEC monolayers.

dHL-60 cells stably co-expressing PECAM-1–GFP and ΔM-N L-selectin–RFP possessed TTTs that were similar to TAPI-0-treated double expressors (expressing WT L-selectin and PECAM-1), with an average TTT of 9 min 18 s (±15 s) (Fig. 6C). In keeping with blocking L-selectin shedding in neutrophils, the migration times were significantly slower when dHL-60 cells were perfused over IL-1β-activated endothelial monolayers [i.e. 7 min 25 s (±12 s)] (Fig. 6D). Although the TTT was significantly slower, the difference was modest when compared with the TTT across TNF-activated HUVEC monolayers (compare Fig 6C and D). In Fig. 4B, the p38 MAPK inhibitor significantly reduced the influence of PECAM-1 clustering in augmenting L-selectin shedding at low doses of PMA stimulation. We therefore supplemented the perfusate with either DMSO or 25 µM SB202198 to determine whether inhibition of p38 MAPKs would impact the TTT for primary human neutrophils. Indeed, neutrophil TTTs were significantly retarded across both TNF- and IL-1β-activated HUVEC monolayers (Fig. 6E,F). However, these TTTs could not be directly attributed to SB202198 specifically blocking L-selectin shedding.

### The contribution of PECAM-1 ITIM in regulating co-clustering of L-selectin and TTT

**Fig. 7.**
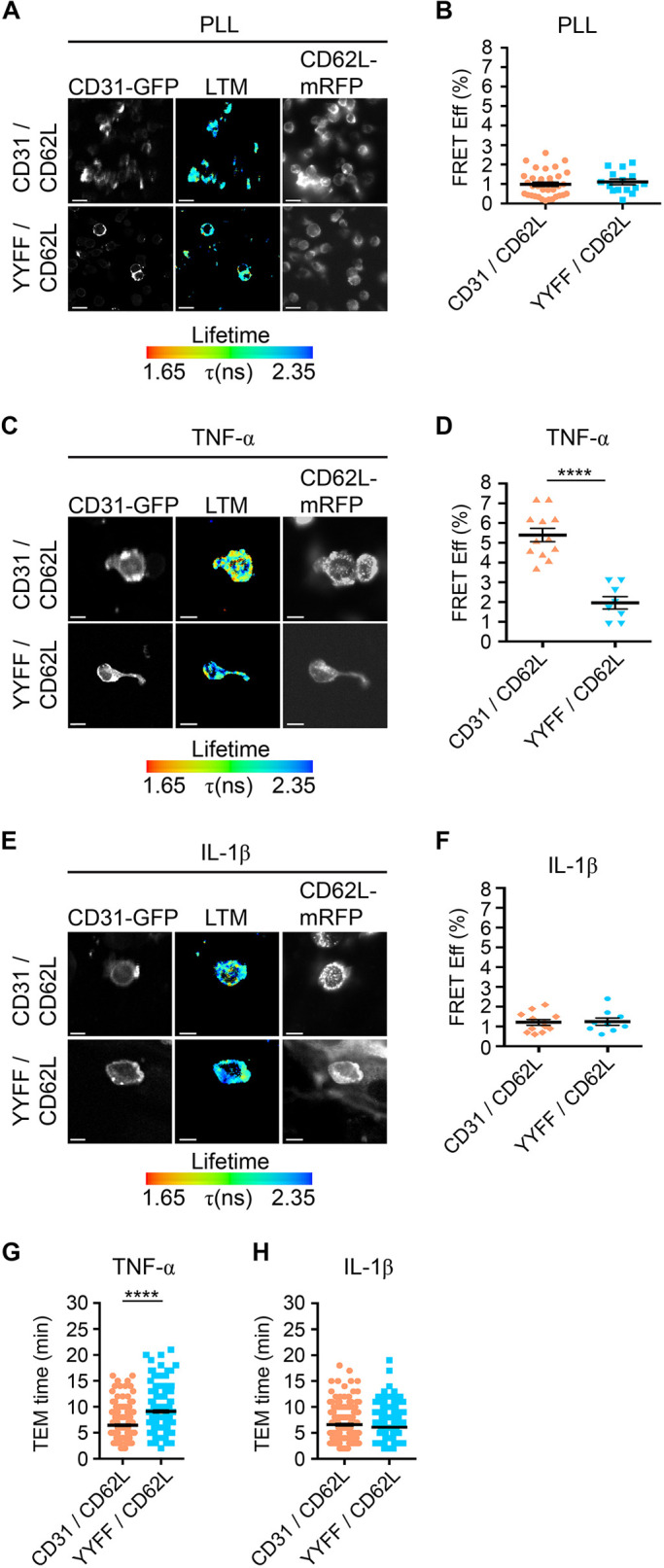
**Mutation of Y663 and Y686 within the PECAM-1 ITIM disrupts co-clustering of L-selectin during TEM and retards TTT across TNF-activated HUVEC monolayers.** (A,B) As in Fig. 2, HL-60 cells expressing L-selectin–RFP and either WT PECAM-1–GFP or the YYFF PECAM-1–GFP ITIM mutant were seeded onto PLL-coated coverslips and subsequently prepared for FLIM. GFP lifetime (LTM) is provided as a pseudocolour scale of blue (long lifetime) to red (short lifetime). (B) The FRET efficiency was quantified and expressed as a percentage, shown in the column scatter graph of mean±s.e.m. (*n*=35 for CD31/CD62L, *n*=15 for YYFF/CD62L). (C–F) dHL-60 cells expressing L-selectin–RFP with either WT PECAM-1–GFP or YYFF PECAM-1–GFP were perfused over either TNF- or IL-1β-activated HUVEC monolayers as previously described in Fig. 2. Following perfusion, cells were fixed and prepared for FLIM analysis for TNF (C,D) or IL-1β (E,F). GFP lifetime (LTM) is provided as a pseudocolour scale of blue (long lifetime) to red (short lifetime). Column scatter graphs contain the mean±s.e.m. measurement from *n*=12 cells for CD31/CD62L, and from *n*=8 and 9 cells for YYFF/CD62L in D and F, respectively, derived from three independent experiments. ****P<0.0001 (one-way ANOVA followed by multiple comparisons corrected for by the Sidak method). Note that the data representing dHL-60 cells stably expressing WT L-selectin and WT PECAM-1 in A–H is taken from previous analyses (specifically from Figs 2 and 5) to compare again the TEM times for YYFF PECAM-1- and WT L-selectin-expressing cells. (G,H) The same cell lines were perfused over either TNF- or IL-1β-activated HUVEC monolayers and TTT was quantified across these monolayers and expressed in the column scatter graphs. Mean±s.e.m. measurements from 180 cells were obtained per group and derived from three independent experiments. ****P<0.0001 (two-tailed unpaired Student's *t*-test with Welch's correction used). Scale bars: 10 μm.

The intracellular tails of L-selectin and PECAM-1 can both interact with ERM proteins (Gamulescu et al., 2003; Ivetic et al., 2002). With respect to L-selectin, ERM binding is absolutely fundamental to antibody mediated (outside-in) clustering (Newe et al., 2019). PECAM-1 also harbours an immunoreceptor tyrosine-based inhibition motif (ITIM) within its relatively longer cytoplasmic domain. Previous work by Florey et al. revealed that two ITIM tyrosine (Y) residues at position 663 and 686 are responsible for modulating TEM of monocyte-like U937 cells *in vitro* (Florey et al., 2010). Given the unidirectional influence of PECAM-1 on driving the co-clustering of L-selectin during TEM, we questioned whether mutation of Y663 and Y868 to non-phosphorylatable phenylalanine (F) residues would impact the co-clustering of L-selectin and therefore retard the TTT. HL-60 cells were therefore engineered to stably express both WT L-selectin–RFP and PECAM-1–GFP containing the Y663F and Y686F mutations (hereafter called YYFF). As with cells expressing WT forms of L-selectin and PECAM-1, seeding HL-60 cells co-expressing WT L-selectin–RFP and YYFF PECAM-1–GFP onto PLL led to negligible increases in FRET efficiency as assessed by FLIM (Fig. 7A,B). FRET/FLIM analysis revealed no significant increases in FRET efficiency between the GFP and RFP tags pairs when these dHL-60 cell lines were perfused over either TNF- or IL-1β-activated HUVECs (Fig. 7C–F). Perfusion of dHL-60 cells over cytokine-activated HUVEC revealed that TTT was reduced significantly in YYFF cells compared to WT cells over TNF-stimulated HUVEC [i.e. 9 min 6 s (±17 s) for YYFF cells versus 6 min 28 s (±14 s) for WT cells] (Fig. 7G). However, the TTT of YYFF cells crossing IL-1β-activated HUVEC monolayers did not differ significantly from dHL-60 cells expressing WT versions of PECAM-1–GFP and L-selectin–RFP (Fig. 7H). These data indicate an absolute requirement of the PECAM-1 ITIM to regulate the co-clustering of L-selectin and TTT of dHL-60 cells crossing TNF- but not IL-1β-activated HUVEC monolayers.

## DISCUSSION

Many investigations have shown neutrophils to be the first immune cell subtype to arrive at a site of extravascular damage or infection. The biological advantage of rapidly deploying neutrophils is to expedite their effector functions and mitigate death to the host (Ley et al., 2018). Our data predicts that blocking L-selectin–PECAM-1 co-clustering in humans would lead to an ∼30% reduction in TTT, which, in theory, could reduce the chance of host survival. Blocking L-selectin shedding in human neutrophils with TAPI-0-like compounds was previously shown not to interfere with TEM (Allport et al., 1997b). It now seems likely that this oversight was down to the lack of close imaging of migration. Beyond TEM, it appears that blocking L-selectin shedding impacts neutrophil chemotaxis *in vivo* (Venturi et al., 2003), suggesting that effector functions may be impaired. Indeed, blocking L-selectin shedding *in vivo* appears to impair bacterial killing *in vivo* (Cappenberg et al., 2019).

**Fig. 8.**
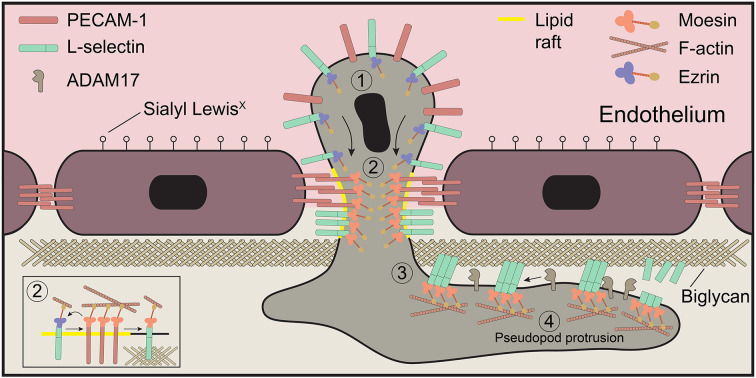
**Working model of L-selectin co-clustering with PECAM-1 during TEM.** Schematic of a neutrophil captured in mid-TEM, depicting the molecular events we hypothesise occur during TEM across TNF-activated endothelial monolayers. (1) L-selectin and PECAM-1 remain unclustered in non-transmigrated uropods. (2) Neutrophil PECAM-1 is clustered in *cis* by endothelial PECAM-1 through homotypic interactions via immunoglobulin domains 1 and 2, which drives the co-clustering of L-selectin. (3) We believe that the clustered L-selectin within the subendothelial space could increase its ability to bind subendothelial glycans, such as biglycan, which could drive one of two signals. (4) Activation of the sheddase ADAM17 to drive the cleavage of L-selectin (e.g. through activation of p38 MAPK; Smolen et al., 2000) and signalling downstream of the clustered L-selectin to drive pseudopod protrusion. We have recently shown in a THP-1 cellular model of monocytes that ezrin and moesin bind to L-selectin in a sequential manner; ezrin to drive signalling through putative interaction with the p85 regulatory subunit of PI3K [not shown here but reviewed elsewhere (Ivetic et al., 2019)] and moesin to facilitate ectodomain shedding. Ectodomain shedding of L-selectin would limit the extent to which leukocytes drive pseudopod protrusion in the subendothelial space, allowing other mechanisms to successfully establish front-back polarity for interstitial chemotaxis.

L-selectin clustering can be promoted in one of two ways – outside-in (e.g. by ligand) and inside-out (e.g. by virtue of an intracellular signal that drives ligand-independent clustering – likely through the reorganisation of the cortical actin-based cytoskeleton). A plenary example of inside-out clustering of L-selectin was demonstrated by exposing T lymphocytes to fever-range temperatures (i.e. 38–41°C), which in turn triggers IL-6-dependent signalling to drive ERK/MEK-mediated L-selectin–cytoskeleton interactions (Chen et al., 2004; Evans et al., 1999; Wang et al., 1998). Chimeric fusion of DNA gyrase to the cytoplasmic tail of L-selectin can promote dimerisation of L-selectin in response to coumermycin treatment (Li et al., 1998). In both these cases, inside-out clustering of L-selectin increases avidity and therefore leukocyte adhesion to immobilised ligands under hydrodynamic shear stress. Indeed, Fig. 1 clearly shows that, in the absence of an L-selectin ligand, antibody-mediated clustering of PECAM-1 is sufficient to drive this co-clustering behaviour. Experimentally, we have shown in THP-1 monocytes that L-selectin clustering occurs in transmigrating pseudopods and not within the non-transmigrated uropods (Rey-Gallardo et al., 2018; Rzeniewicz et al., 2015). Our data here would suggest that the co-clustering of L-selectin with PECAM-1 could drive L-selectin into clusters, to increase its avidity for adhesion to other non-sialyl Lewis-x ligands (such as biglycan) within the subendothelial space (see Fig. 8). Heparan sulfate proteoglycans (HSPGs) are reportedly enriched in the basolateral aspect of the endothelium (Stoler-Barak et al., 2014). Furthermore, we and others have shown that the HSPGs biglycan and versican are credible ligands for L-selectin (Celie et al., 2009; Kawashima et al., 2000, 1999; Kitaya and Yasuo, 2009; Rzeniewicz et al., 2015). We believe that signals downstream of L-selectin–HSPG binding would be sustained, as the absence of shear stress in the subendothelial space cannot readily readily break the L-selectin–HSPG bonds. The signals propagated in this region would therefore lead to as-yet-unidentified signals driving pseudopod protrusion in TEM. L-selectin-dependent signalling is potentially self-limiting, as clustering of L-selectin on neutrophils is sufficient to drive its ectodomain shedding (Palecanda et al., 1992).

Our hypothetical model of how PECAM-1 and L-selectin corroborate to drive pseudopod protrusion in TEM is summarised in Fig. 8. Although we know that monocyte-derived (U937 cell) PECAM-1 enters lipid rafts during TEM (Florey et al., 2010), we speculate that L-selectin and PECAM-1 may co-cluster specifically within lipid rafts during TEM. Within this microdomain, it is possible that the exchange of ezrin for moesin on L-selectin can occur. We have shown that serine phosphorylation of the L-selectin tail at position S364 can abrogate ERM binding *in vitro* (Newe et al., 2019). And that clustering of L-selectin in Jurkat T-cells can drive cytoplasmic tail phosphorylation and subsequent enrichment into lipid rafts (Phong et al., 2003). These independent observations would imply that the transient and sequential phosphorylation of L-selectin and PECAM-1 cytoplasmic tails could drive their accumulation into lipid rafts – wherein ERM exchange could ensue. We also noticed that antibody-mediated clustering of PECAM-1 led to a 22% reduction in L-selectin expression, likely through internalisation and not shedding. Currently, we are not sure whether this is a caveat to the experimental procedure, or whether internalisation of L-selectin actually occurs during TEM.

The Akt family – a faithful biochemical readout for PI3K activation – was robustly phosphorylated following PECAM-1 clustering. An early observation showed that PECAM-1 can associate (directly or indirectly) with the p85 regulatory subunit of PI3K (Pellegatta et al., 1998). Whether co-clustering of L-selectin with PECAM-1 is required for PI3K activation has yet to be tested. We also observed an increase in phospho-JNK following PECAM-1 clustering (Fig. 4A). SP600125-mediated shedding of L-selectin in neutrophils suggests that JNK may be constitutively active to protect L-selectin from shedding when PECAM-1 is clustered. Activation of JNK would therefore be in conflict with synchronous activation of p38 MAPK, which primes L-selectin for shedding (Killock and Ivetic, 2010). It is tempting to speculate that JNK activation could be transient, whereas activation of p38 MAPK is more sustained. Therefore, protection of L-selectin shedding (through JNK activation) in early TEM could ensure signals downstream of L-selectin can contribute to pseudopod protrusion. Upon JNK inactivation (e.g. when neutrophils begin to enter the subendothelial space), p38 MAPK activity may then preside to prime L-selectin for ectodomain shedding in late TEM. L-selectin clustering in neutrophils can also prime neutrophil effector function (Smolen et al., 2000). Whether these effects of L-selectin are enhanced when PECAM-1 is clustered is yet to be determined.

Another compelling finding in this study is the cytokine-specific contribution of L-selectin–PECAM-1 co-clustering in regulating TTT specifically across TNF-, but not across IL-1β-, activated HUVEC monolayers. Our data imply that neither Ig domains 1 and 2 of neutrophil PECAM-1, nor the ITIM residues Y663 and Y686, are required for optimal TEM across IL-1β-activated HUVEC monolayers. Currently, it is not known whether IL-1β can upregulate a unique (glyco)protein(s) to co-operate with PECAM-1 and alleviate the need for Y663 and Y686 and Ig domains 1 and 2 for optimised TEM. A comparative proteomic analysis of (glyco)protein expression in HUVECs, following stimulation with either IL-1β or TNF, could facilitate a better understanding of whether an additional factor may be involved in driving PECAM-1-dependent TEM across IL-1β-activated HUVECs. A recent report shows that neutrophils harvested from synovial fluid aspirates of patients with chronic inflammatory arthritis are L-selectin positive (Bjorkman et al., 2018); in contrast, neutrophils harvested from acutely formed skin blisters are L-selectin negative (Bjorkman et al., 2018). Whether this observation implies neutrophils are crossing a higher proportion of IL-1β-activated post-capillary venules in chronically inflamed joints (compared to post-capillary venules in acutely inflamed skin blister) would be an interesting topic to investigate further. If this is indeed the case, then quantifying the extent of L-selectin-positive neutrophils in a given histopathological section may act as a useful biomarker for the predominance of TNF or IL-1β within a given histopathological specimen.

## MATERIALS AND METHODS

### Reagents and antibodies

All reagents were purchased from Sigma-Aldrich, unless otherwise stated. TNF protease inhibitor-0 (TAPI-0) was purchased from Santa Cruz Biotechnologies and dissolved in DMSO. Anti-L-selectin antibodies, DREG56 (mouse monoclonal IgG1 κ) and Alexa Fluor^®^ 647-conjugated DREG56, were purchased from Santa Cruz Biotechnologies (cat. #sc-18851). Anti-human PECAM-1 (HEC7, mouse monoclonal IgG2a, Thermo Fisher Scientific: cat. #MA3100) and IgG isotype controls were purchased from Thermo Fisher Scientific. Anti-human PECAM-1 (sheep polyclonal IgG) was purchased from R&D Systems (cat. #AF806-SP) and targets the extracellular domain of PECAM-1. HEC7 is a mouse IgG2a monoclonal antibody that specifically targets immunoglobulin domains 1 and 2 of human PECAM-1. LAM1-14 mouse monoclonal IgG1 is a non-function-blocking antibody for human L-selectin and was kindly provided by Thomas Tedder (Duke University, USA) (Spertini et al., 1991). DREG56 specifically targets the lectin domain of human L-selectin and is a function-blocking antibody. Note that both HEC7 and LAM1-14 are both mouse monoclonals and could therefore not be used for clustering and secondary antibody labelling for confocal microscopy (as in Fig. 1C,D). Instead, sheep anti-human PECAM-1 antibody (R&D Systems) was used.

Antibody dilutions for immunofluorescence and FACS were as follows: LAM1-14 was used at 1:300 dilution for immunofluorescence staining (from a 1 μg/ml stock); anti-human PECAM-1 sheep polyclonal antibody was used at 1:200 (from a 1 μg/ml stock); DREG56 was used at a dilution of 1:200 (from a 200 μg/ml stock); and HEC7 monoclonal antibody was used at 1:1000 dilution (from a 1 μg/ml stock).

Antibodies used for clustering cell adhesion molecules in Fig. 1A were: CD43 (DFT-1) mouse monoclonal (Santa Cruz Biotechnologies, cat. #sc-6256; 200 μg/ml stock, dilution 1:100); JAM-A (J10.4) mouse monoclonal (Santa Cruz Biotechnologies, cat. #sc-53623; 200 μg/ml stock, dilution 1:100); CD31 (JC70A) mouse monoclonal (Agilent, cat. #M082329-2; 1 mg/ml stock, dilution 1:500); CD44 rabbit polyclonal (Santa Cruz Biotechnologies, cat. #sc-7946; 200 μg/ml stock, dilution 1:100); and PSGL-1 (KPL1) mouse monoclonal (Santa Cruz Biotechnologies, cat. #sc-13535; 200 μg/ml stock, dilution 1:100). Secondary antibodies used were purchased form Thermo Fisher Scientific: Alexa Fluor^®^ 633-conjugated goat anti-mouse (cat. #A-21052); Alexa Fluor^®^ 633-conjugated goat anti-rabbit (cat. #A-21070); Alexa Fluor^®^ 633-conjugated donkey anti-sheep (cat. #A-21100); Alexa Fluor^®^ 647-conjugated donkey anti-mouse (cat. #A32787); Alexa Fluor^®^ 647-conjugated donkey anti-rabbit (cat. #A32795); Alexa Fluor^®^ 488-conjugated goat anti-mouse (cat. #A-11001); Alexa Fluor^®^ 488-conjugated goat anti-rabbit (cat. #A-11008); Alexa Fluor^®^ 488-conjugated donkey anti-mouse (cat. #A-21202); Alexa Fluor^®^ 488-conjugated donkey anti-rabbit (cat. #A-21206); and Alexa Fluor^®^ 546-conjugated donkey anti-mouse (cat. #A10036). All secondary antibodies were provided at a stock concentration of 1 mg/ml and were used at a dilution of 1:300. Goat secondary antibodies were used for Fig. 1A,B. Donkey secondary antibodies were used for Fig. 1D and Fig. 3.

### Human umbilical vein endothelial cells

Human umbilical vein endothelial cells (HUVECs) were purchased from Lonza and expanded in fibronectin-coated 75 cm^2^ flasks (10 μg/ml in PBS) in a 37°C/5% CO_2_ incubator until passage 4. HUVECs were grown using Lonza EGM-2 growth medium supplemented with human epidermal growth factor (hEGF), vascular endothelial growth factor (VEGF), R3-insulin-like growth factor (R3-IGF-1) and human fibroblast growth factor-β (hFGF-β). HUVECs were detached from flasks using trypsin-EDTA under conditions of 37°C/5% CO_2_ for 3–5 min.

### Culture and differentiation conditions for THP-1 cells and HL-60 cells

THP-1 cells were purchased from ATCC and cultured as previously published (Newe et al., 2019; Rey-Gallardo et al., 2018; Rzeniewicz et al., 2015). HL-60 cells stably expressing CXCR2 were kindly provided by Prof. Ann Richmond (Vanderbilt University, USA) (Sai et al., 2006) and tested for contamination, but not recently authenticated. Cells were grown in HL-60 medium [RPMI (Gibco); 10% FCS; 1% penicillin streptomycin (Gibco)] and kept to densities of 0.5×10^6^ per ml. Differentiation of HL-60 cells (dHL-60) was performed by culturing HL-60 cells at a density of 1×10^6^ cells per ml in 1.3% dimethyl sulfoxide (DMSO).

### Fluorescence-activated cell sorting

Cell lines were sorted by dedicated support staff at the Flow Cytometry Facility located in the Clinical Research Centre at Guy's and St Thomas’ NHS Foundation Trust and King's College London, 15th floor, Tower Wing, Guy's Hospital, Great Maze Pond, London, SE1 9RT.

### Mutagenesis of PECAM-1 and L-selectin

Human PECAM-1 cDNA amplified from pWPT lentiviral plasmid, with open reading frames (ORFs) of WT and Y663, 686F (YYFF) mutant PECAM-1 (kindly donated by Oliver Florey, Babraham Institute, UK; Florey et al., 2010) were subcloned into pHR'SIN-SEW lentiviral vector containing the ORF GFP 3′ to the multiple cloning site. The primers used to amplify PECAM-1 were engineered to contain AscI and KpnI restriction sites at the 5′ and 3′ ends, respectively: PECAM-1 forward primer, 5′-GAGAGAGGCGCGCCATGCAGCCGAGGTGGG-3′ and PECAM-1 reverse primer, 5′-CTCTCTGGTACCAGTTCCATCAAGGGAGCC-3′.

Sanger sequencing by Source BioScience (Nottingham, UK) ensured correct insertion of the PECAM-1 cDNA, and that no unexpected mutations or frame shifts occurred during the subcloning procedure. PCR-based mutagenesis of PECAM-1 was conducted according to the manufacturer's instructions (Agilent Technologies). The primers used for the generation of PECAM-1 small nucleotide polymorphisms (SNPs) are listed below. Following mutagenesis, all constructs were fully sequenced to confirm mutations had been faithfully incorporated. PECAM-1 in either its WT form or containing the tyrosine to phenylalanine mutation at positions 663 and 686 (YYFF) was mutated as described by Florey et al. (2010), and subcloned into lentiviral vectors (pHR'SIN-SEW, see below).

WT and mutant L-selectin open reading frames were subcloned into pHR'SIN-SEW lentiviral vectors as previously described (Rzeniewicz et al., 2015). The lentiviral backbone vectors were provided by Adrian Thrasher, Institute of Child Health, University College London, London, UK, which carried either enhanced GFP (hereafter referred to as GFP) or monomeric mCherry (referred to as RFP in this work) as C-terminal tags for PECAM-1 and L-selectin, respectively.

### Generation of HL-60 cell lines

HL-60 cells stably expressing CXCR2 (kind gift from Prof. Ann Richmond, Vanderbilt University, USA; Sai et al., 2006) were infected with lentiviral particles containing the pHR'SIN-SEW vector and open reading frames for GFP, L-selectin-RFP or PECAM-1-GFP. More information on the lentiviral vectors used can be found in our previous reports (Burns et al., 2010; Rey-Gallardo et al., 2018; Rzeniewicz et al., 2015). All HL-60 cells were prepared to a density of 1×10^6^ cells per ml and subsequently infected with a multiplicity of infection of (MOI) 10 for each construct. After infection, cells were propagated until sufficient numbers were obtained for cell sorting at the dedicated flow cytometry facility at the National Institute for Health Research (NIHR) Biomedical Research Centre (Guy's Hospital, London). Sorted cells were analysed by FACS to validate equal expression between cell lines, as shown in Fig. S5.

### Antibody-mediated clustering of L-selectin or PECAM-1

Freshly isolated neutrophils were labelled with 1 µg/ml of DREG-56 (a mouse IgG1κ monoclonal antibody directed at the lectin domain of human L-selectin), HEC7 (mouse IgG2a monoclonal antibody targeting immunoglobulin domains 1 and 2 of human PECAM-1) or anti-human PECAM-1 antibody (sheep polyclonal IgG) for 30 min on ice. Note that both HEC7 and LAM1-14 are both mouse monoclonals and could therefore not be used for clustering and secondary antibody labelling for confocal microscopy (as in Fig. 1). Instead, sheep anti-human PECAM-1 antibody (R&D Systems) was used. All labelling procedures were conducted in Fc-receptor block (Miltenyi Biotec) and 33.3% fetal calf serum to ensure primary or secondary antibodies were not targeting the Fc-receptors. Labelling steps were followed by two wash steps in phosphate-buffered saline (PBS) to remove any excess antibody. Subsequent labelling with 10 µg/ml of either anti-mouse or anti-sheep secondary antibody conjugated to either Alexa Fluor^®^ 488 or 633 was used for microscopy (post-fixation) and overnight staining or for FACS. After washing off excess secondary antibody, cells were resuspended in neat RPMI and then plated onto poly-L-lysine-coated coverslips for the indicated times. Cells were fixed in 4% (v/v) paraformaldehyde (PFA) in PBS without Ca^2+^ and Mg^2+^. Specimens were further prepared for confocal, FRET/FLIM or inverted structural illumination microscopy (iSIM). Clustering experiments were performed at 37°C for 25 min.

### Isolation of primary human neutrophils and CD14-positive monocytes

Primary human neutrophils were isolated from whole blood of healthy volunteers at the King's College Hospital Clinical Research Facility, in accordance with approval by the local research ethics committee at King's College London.

Freshly drawn blood (25 ml) was layered onto 15 mL of Histopaque-1077 and centrifuged at 186 ***g*** for 30 min at room temperature and pressure (RTP) with acceleration/deceleration set to 1/1, respectively. Three layers above a bottom layer of red blood cells formed after centrifugation: a yellow plasma layer; a buffy coat layer; and a clear Histopaque-1077 layer. The buffy coat was retained specifically for further isolation of CD14-positive monocytes, according to manufacturer's instructions (CD14 negative selection kit; Miltenyi Biotec), as we have described previously (Rey-Gallardo et al., 2018; Rzeniewicz et al., 2015). We have also previously published the isolation of neutrophils from whole blood in great detail (Killock and Ivetic, 2010).

### MAPK array of primary human neutrophils following PECAM-1 clustering

A Proteome Profiler^TM^ Human Phospho-MAPK Array Kit (R&D Systems) was used to assess which family of MAPKs became phosphorylated (and therefore activated) in response to PECAM-1 clustering. Approximately 10^7^ freshly isolated primary human neutrophils were left untreated or incubated with 1 μg/ml mouse anti-CD31 HEC7 on ice for 30 min in the presence of Fc Receptor block and 33% FCS, followed by clustering with a secondary anti-mouse antibody (see exact details in ‘Antibody-mediated clustering of L-selectin or PECAM-1’ section above). Neutrophils were subsequently incubated at 37°C for 20 min, harvested by centrifugation, and the pellet lysed with a proprietary lysis buffer. Following centrifugation at 13,000* **g*** for 15 min to clarify the lysate, samples were incubated with the membrane-immobilised antibody array in accordance with the manufacturer's protocol. Membranes were left overnight at 4°C to incubate with gentle agitation. The following day, membranes were washed in PBS, blocked and probed (all at RTP) with a cocktail of biotinylated phospho-specific antibodies. Membranes were washed prior to incubation with streptavidin-horseradish peroxidase and a chemiluminescent reagent mix, before development by exposure to X-ray film. Images of the array were acquired by scanning the X-ray films and visualisation achieved using a GS-800 BioRad gel scanner.

### Parallel plate flow chamber assays

All flow assays were conducted using the Glycotech platform. The experimental setup has been published in previous reports from our laboratory (Newe et al., 2019; Rey-Gallardo et al., 2018; Rzeniewicz et al., 2015). Importantly, HUVECs were grown to confluence of 35 mm circular coverslips (thickness=1.0; purchased from Menzel Glaser, VWR), pre-coated with 10 µg/ml bovine fibronectin (diluted in PBS) and stimulated overnight (18 h) with either 10 ng/ml recombinant human TNF (R&D Systems) or 10 ng/ml recombinant human interleukin-1β (IL-1β, Sino Biological^®^). All flow assays were performed at 1.5 dynes/cm^2^. Cells exposed to the sheddase inhibitor TAPI-0 or the p38 MAPK inhibitor SB202190 were preincubated for 20 min prior to flow assays. Both TAPI-0 and SB202190 are reversible inhibitors, so leukocytes were exposed to the inhibitor beforehand, and the perfusate was also supplemented in the medium (10 µM for TAPI-0 and 25 µM for SB202190). HUVECs were exposed to TAPI-0 but not SB202190 prior to flow chamber assays to limit the impact of the inhibitor specifically towards leukocytes and not endothelial cells. Control experiments were performed with the carrier, DMSO, as previously described (Rzeniewicz et al., 2015).

An Olympus IX81 epifluorescence microscope was used for all acquisitions of live flow assays, using the same bright field (for phase contrast) and fluorescence intensity settings (for GFP and RFP). A 10× objective lens was used (numerical aperture=0.3) for all acquisitions. All experiments were conducted under stable thermal setting of 37°C, by virtue of a bespoke heated chamber surrounding the microscope stage. Volocity software was used to control image acquisitions, taken by a Hamamatsu C10600 ORCA-R2 digital camera. Each frame was taken every 4 s for primary human neutrophils (phase only) and every 50 s for dHL60 cells (phase and fluorescence).

In assays where confocal microscopy or FRET/FLIM analysis is used, specimens were fixed immediately in 4% PFA and prepared for each respective technique as previously described (Newe et al., 2019; Rey-Gallardo et al., 2018; Rzeniewicz et al., 2015).

### Calculating the time taken for transendothelial migration

Imaging of transmigrating cells was performed using time-lapse epifluorescence microscopy – where HL-60 cells would be serially imaged in phase and then fluorescence channels (to detect either PECAM-1–GFP or L-selectin–RFP). Images were acquired once every 4 s for primary human neutrophils (phase only) and every 50 s for dHL60 cells (phase and fluorescence). Using the phase channel, cells were tracked from the moment of adhesion to HUVECs from flow to the moment they have undergone full transendothelial migration. The transition from adhesion to complete TEM was scored as the change from being ‘phase bright’ to ‘phase dark’ – as shown in the stills 2–10 represented in Fig. 5A and Movies 2 and 3 for primary human neutrophils and dHL-60 cells, respectively.

### Ectodomain shedding assays using flow cytometry and confocal microscopy

Primary human neutrophils were left untreated or incubated with HEC7 monoclonal antibody at 1 μg/ml for 30 min in blocking buffer [containing human Fc Receptor block (Miltenyi Biotec) and 33.3% FCS in PBS] at 37°C. Excess (unbound) HEC7 antibody was subsequently washed through centrifugation and resuspension of the pellet with RPMI-1640 medium containing Fc receptor block and FCS. Clustering of PECAM-1 in a subset of cells was performed by labelling cells on ice with Alexa Fluor^®^ 488 donkey anti-mouse-IgG antibody at 10 μg/ml for 5 or 30 min at 37°C to drive L-selectin clustering. After washing off excess secondary antibody in HL-60 medium and blocking agents at room temperature, neutrophils were incubated in HL-60 medium containing phorbol-12′-myristate-13′-acetate (PMA) at various concentrations, for 30 min at 37°C. Neutrophils were then immediately transferred to ice-cold HL-60 medium prior to washing steps and labelling with DREG56 directly conjugated to Alexa Fluor^®^ 647 for 30 min, at 4°C. Neutrophils labelled with just HEC7 were also subsequently labelled with DREG56 conjugated to Alexa Fluor^®^ 647 for 30 min at 4°C. In some experiments, neutrophils were preincubated for 10 min with either 10 µM TAPI-0 or 25 µM SB202190 at 37°C prior to secondary antibody labelling. In each case, the inhibitor remained present during incubation with secondary antibody. Cells not challenged with secondary antibody or drugs were taken through the same procedure containing just carrier (DMSO) and/or non-specific isotype-matched antibody.

For monitoring L-selectin shedding induced by TEM (as in Fig. 3), neutrophils were prepared for parallel plate flow chamber assays as described above. After 20 min of perfusion, specimens were rapidly fixed in 4% PFA and subsequently prepared for immunostaining. Specimens were washed three times in PBS before permeabilisation on ice for 3 min in PBS containing 0.1% (v/v) NP-40 substitute, and blocked for 30 min at room temperature using human Fc Receptor block (Miltenyi Biotec) and 33.3% FCS in PBS. The anti-L-selectin monoclonal antibody, LAM1-14, was used to detect L-selectin expression in neutrophils that had exclusively entered the subendothelial space (as shown in Fig. 5D,E).

Following 20 min of perfusion, under wide-field microscopy, ∼95% of all neutrophils had fully transmigrated and adopted a flat phase dark phenotype (see Movie 2). LAM1-14-positive staining of fully transmigrated neutrophils was assessed using the line intensity tool in Volocity software. Two diametrically opposed lines of equal length were created to bisect each cell that was under analysis (see Fig. 3C). The pixel intensity values of the two lines were averaged, and these mean intensity values were subsequently collected for every cell analysed. The total of fluorescence intensity values was then divided by the number of cells analysed across three independent experiments (i.e. ∼420 neutrophils per treatment group). The fluorescence intensity values for TAPI-0 treatment were normalised to represent maximal (100%) retention of L-selectin expression after TEM. This value was then used to determine the percentage loss of L-selectin expression in the DMSO treatment group.

### Inverted structural illumination microscopy

Images were acquired at 100× magnification (1.49 NA) on a vt-iSIM (a high-speed super resolution imaging system) with Hamamatsu Flash4.0 sCMOS camera and operated by Nikon NIS elements software. The vt-iSIM lasers used were at wavelengths 488 nm and 642 nm. *Z*-stacks in 3D were obtained at a resolution of 0.12 μm. Post-acquisition analysis involved deconvolution, which was performed in NIS elements, and applying the Richardson–Lucy algorithm at a mounting medium refractive index of 1.372. NIS elements software was used to obtain Manders’ overlap coefficiencies (M2 Coeff) of L-selectin and PECAM-1 signal overlap by thresholding signals to eliminate background. Coefficients were obtained for three distinct optical slices representing the apex (top), middle and base of primary human neutrophils adhering to PLL-coated coverslips.

#### Confocal microscopy and Manders’ overlap coefficiencies obtained by Volocity

Images of THP-1 cells and primary human monocytes were acquired using a Leica TCS SP5 microscope and a Leica oil immersion 63× objective lens (NA 1.4). Optical sections were acquired at a resolution of 0.72 µm. Images acquired by confocal microscopy were analysed by Volocity software to obtain the Manders’ overlap coefficiency. Signal intensities were set above background for both green and blue channels, which respectively corresponded to PECAM-1 and L-selectin, using the voxel spy tool. Instrument parameter settings were maintained in all of the image acquisitions. Complete (100%) overlap between signals is designated as having a value of 1. All neutrophil images were acquired by structured illumination microscopy (see iSIM section). M2 Coeff were acquired using NIS elements software.

### Western blotting of PECAM-1 from HUVEC monolayers

Confluent monolayers of HUVECs in six-well plates were stimulated for 17 h with 10 ng/ml TNF, IL1-β, or no stimulation. Cells were subsequently lysed in 100 µl 2× protein loading buffer supplemented with 200 mM DTT and 100 units/ml benzonase nuclease before boiling at 100°C for 5 min. HUVEC lysates were loaded 25 µl per sample into a NuPage 4–12% Bis-Tris gel. Proteins were separated by SDS-PAGE using 1× NuPAGE MES SDS running buffer for 1 h at 150 V. Proteins were transferred to a nitrocellulose membrane, using XCell II Blot Module (Thermo Fisher Scientific), over 2.5 h at 30 V. Membranes were blocked for 1 h in 5% milk powder in Tris-buffered saline (TBS) before incubation with primary antibodies: sheep anti-human CD31 (1:200 dilution, R&D Systems AF806) and mouse anti-human β-actin (1:4200 dilution) at 4°C overnight. Membranes were washed three times for 5 min in TBS and 0.1% (v/v) Tween-20 (TBST) followed by incubation with secondary antibodies – donkey anti-goat-IgG (1:15,000, LiCOR IRDye^®^ 680CW) or donkey anti-mouse-IgG (1:15,000, LiCOR IRDye^®^ 800CW) for 1 h at room temperature. TBST washes were repeated, followed by a final wash with TBS for 5 min. Western blots were developed using the Odyssey CLx Near-Infrared Fluorescence Imaging System and Image Studio software was used to quantify relative band intensities.

### Fluorescence resonance energy transfer and fluorescence lifetime imaging microscopy

dHL-60 cells stably expressing L-selectin–RFP and PECAM-1–GFP (either WT or mutant forms) were perfused over cytokine-activated (either IL-1β or TNF) HUVEC monolayers and fixed at the point that the majority of cells were captured in mid-TEM. Specimens were counterstained with anti-PECAM-1 antibody to determine the position of endothelial cell junctions. An example of a leukocyte captured in mid-TEM is provided in Fig. 3A, where junctional staining of PECAM-1 is breached and where regions of the transmigrating cell are above and below the endothelium. Given that the enrichment of PECAM-1 is at cell-to-cell junctions, FRET was measured and quantified exclusively at breached junctions and readings were taken neither exclusively above (adherent and non-transmigrated) nor exclusively below (fully completed TEM) the endothelium.

FLIM measurement of FRET was performed with a multiphoton microscope system as described previously (Newe et al., 2019; Rey-Gallardo et al., 2018; Rzeniewicz et al., 2015). A Nikon TE2000E inverted microscope, combined with an in-house scanner and Chameleon Ti:Sapphire ultrafast pulsed multiphoton laser (Coherent Inc.), was used for excitation of GFP (at 890 nm). Fluorescence lifetime imaging capability was provided by time-correlated, single-photon counting electronics (SPC 700; Becker & Hickl). A 40× objective (NA 1.3) was used throughout (CFI60 Plan Fluor; Nikon), and data were acquired at 500±20 nm through a bandpass filter (35-5040; Coherent Inc.). Acquisition times of ∼300 s at low excitation power were used to achieve sufficient photon statistics for fitting; avoiding either pulse pile-up or significant photobleaching. Data were analysed as previously described (Parsons et al., 2008). The FRET efficiency is related to the molecular separation of donor and acceptor and the fluorescence lifetime of the interacting fraction by:
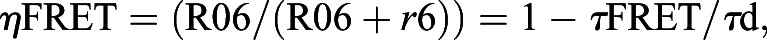
where *η*FRET is the FRET efficiency, R0 is the Förster radius, *r* is the molecular separation, τFRET is the lifetime of the interacting fraction and τd is the lifetime of the donor in the absence of an acceptor. The donor-only control is used as the reference against which all of the other lifetimes are calculated in each experiment. τFRET and τd can also be taken to be the lifetime of the interacting fraction and non-interacting fraction, respectively. Quantification of FRET was made from all pixels within each cell that was analysed. All image collection and data analysis were performed using TRI2 software (developed by Paul Barber, Gray Cancer Institute, London, UK).

## Supplementary Material

Reviewer comments
