# Supplementary material for: L-selectin regulates human neutrophil transendothelial migration
Source: J Cell Sci. 2021 Feb 8;134(3):jcs250340. doi: 10.1242/jcs.250340 (PMC7888707; doi:10.1242/jcs.250340)
Supplement: Reviewer comments [file joces-134-250340_review_history.pdf]

Journal of Cell Science

L-selectin regulates human neutrophil transendothelial migration

Izajur Rahman, Aida Collado Sánchez, Jessica Davies, Karolina Rzeniewicz, Sarah Abukscem, Justin Joachim, Hannah L. Hoskins Green, David Killock, Maria Jesus Sanz, Guillaume Charras, Maddy Parsons and Aleksandar Ivetic
DOI: 10.1242/jcs.250340

Editor: Arnoud Sonnenberg

Review timeline
Original submission: 18 June 2020
Editorial decision: 16 July 2020
First revision received: 20 November 2020
Accepted: 3 December 2020

Original submission

First decision letter

MS ID#: JOCES/2020/250340

MS TITLE: L-selectin regulates human neutrophil transendothelial migration

AUTHORS: Izajur Rahman, Aida Collado Sanchez, Jess Davies, Karolina Rzeniewicz, Sarah Abukscem, Justin Joachim, Maria Jesus Sanz, Guillaume Charras, Maddy Parsons, and Aleksandar Ivetic
ARTICLE TYPE: Research Article

We have now reached a decision on the above manuscript.

To see the reviewers' reports and a copy of this decision letter, please go to: https://submit-jcs.biologists.org and click on the 'Manuscripts with Decisions' queue in the Author Area. (Corresponding author only has access to reviews.)

As you will see, the reviewers raise a number of substantial criticisms that prevent me from accepting the paper at this stage. They suggest, however, that a revised version might prove acceptable, if you can address their concerns. If you think that you can deal satisfactorily with the criticisms on revision, I would be pleased to see a revised manuscript. We would then return it to the reviewers.

We are aware that you may currently be unable to access the lab to undertake experimental revisions. If it would be helpful, we encourage you to contact us to discuss your revision in greater detail. Please send us a point-by-point response indicating where you are able to address concerns raised (either experimentally or by changes to the text) and where you will not be able to do so within the normal timeframe of a revision. We will then provide further guidance. Please also note that we are happy to extend revision timeframes as necessary.

Please ensure that you clearly highlight all changes made in the revised manuscript. Please avoid using 'Tracked changes' in Word files as these are lost in PDF conversion.

I should be grateful if you would also provide a point-by-point response detailing how you have dealt with the points raised by the reviewers in the 'Response to Reviewers' box. Please attend to all of the reviewers' comments. If you do not agree with any of their criticisms or suggestions please explain clearly why this is so.

© 2020. Published by The Company of Biologists under the terms of the Creative Commons Attribution License (https://creativecommons.org/licenses/by/4.0/).

Reviewer 1

Advance summary and potential significance to field

The paper describes a role for clustering of L-selectin by PECAM during leukocyte transmigration. The clustering of L-selectin favours shedding and increases the rate of transmigration across TNF-alpha stimulated but not IL-1 stimulated HUVEC monolayers. Shedding deficient L-selectin or mutations in PECAM reduce the rate of TNFalpha dependent but not IL-1 dependent transmigration. This is the first time a role for P-selectin in transmigration has been described.

Comments for the author

Although an interesting finding the differences between the mechanism of transmigration across IL-1 vs TNF alpha stimulated HUVEC is unclear and this is central to understanding the process; does homotypic PECAM interactions happen with both cytokine stimulation? What is different about the process occurring in IL-1, why is clustering not required in this situation. Does this argue that overall clustering isn't that important? How specific is the effect of PECAM clustering given it appears clustering CD43 can have a similar role. There is no evidence for a difference in neutrophil function following transmigration via a shedding dependent (TNF alpha) or non-dependent (Il-1) route. Does shedding play an important role in governing neutrophil function like it does in monocytes?
There is no evidence that this role is important in physiological settings.

Overall the statement that L-selectin regulates neutrophil transendothelial migration appears true but only in specific circumstances.

Comments in detail
Generally the data presented throughout would be better presented if shown as individual data points to get a better idea of data spread and variability.

Fig.1
A-There is a clear difference in L-selectin clustering between cross-linking antibodies, however the fact CD43 can cluster L-selectin to the same magnitude to PECAM surely indicates this is not a specific interaction.

How does the FRET efficiency vary with shape? All the images of cells with high FRET efficiency appear more rounded.

B,C and D- There does appear to be greater overlap between PECAM and L-selectin however in some situations the PECAM and L-selectin localisation appears to change to larger vesicular structures see C (PECAM X-link). Given the secondary crosslinker is added at 37C for 25 min, can you rule out co-internalisation? Some of the punctae in D also appear to be inside the cell.

Fig.2
This figure shows a convincing difference between IL-1 and TNFalpha treated cells.
The figure should also provide evidence that the cells are undergoing TEM (which presumably the authors already have eg. brightfield microscopy).

Fig.3
This figure demonstrates there is an effect of blocking L-selectin shedding on levels of L-selectin in TNF alpha but not IL-1. An image of L-selectin levels before transmigration would have helped interpretation of this data as would co-staining with PECAM. Are there any changes in PECAM levels following IL-1 or TNF alpha treatment?

Fig.4
This figure provides evidence that PECAM crosslinking can increase PMA mediated shedding in a manner that is dependent on MAPK. This experiment seeks to replicate the clustering and shedding that the authors hypothesise occurs as the cells transmigrate. However the clustering agent, the means to induces shedding and the kinetics of the experiment all differ from what is thought to

© 2020. Published by The Company of Biologists under the terms of the Creative Commons Attribution License (https://creativecommons.org/licenses/by/4.0/).

occur during leukocyte transmigration. So although there is clear experimental differences shown there is no evidence that it mimics anything physiological.

Fig.5
B and C This figure provides evidence that, in a cell line, expression of PECAM and L-selectin together expedites transmigration following TNF alpha treatment however PECAM alone is sufficient with IL-1 treatment. Blots demonstrating equal level of expression of each of the constructs would strengthen this data.

Fig.6
C and D Shedding resistant L-selectin reduces TM time in TNFalpha and to some extent IL-1 treated cells. C the data is incomplete and the cells expressing delta M-N and CD31 should also be treated with TAPI, presumably it should have no effect. D There is no data for the effect of TAPI treatment or cells expressing L-selectin alone. C and D would have benefited from western blots to demonstrate similar expression levels of proteins.

Fig.7
This figure demonstrates that clustering is dependent on the two ITIM residues in the intracellular domain of PECAM in TNF alpha treated cells but not IL-1 treated cells. These residues are necessary for a normal rate of transmigration in TNF alpha but not IL-1. Western blots would improve the figure giving an idea of receptor expression. In figure 5 C PECAM alone was sufficient to increase the rate of transmigration so the lack of an effect on 7D argues that the ITIM residues are unnecessary for IL-1 dependent transmigration.

Fig.8
In TNF alpha stimulated cells. Currently the data supporting a homotypic interaction of PECAM driving L-selectin clustering is only supported by the PECAM blocking antibody. There is no evidence from perturbing the other, endothelial side. There is no evidence that clustering of L-selectin is maintained post transmigration this could be addressed with the shedding resistant L-selectin to uncouple the two events.

Reviewer 2

Advance summary and potential significance to field

The authors investigated the contribution of L-selectin on transendothelial migration of human neutrophils. They identify PECAM-1 L-selectin co-clustering and L-selectin shedding through ADAM17 as critical processes during TEM. Interestingly, these mechanisms seem to be stimulus specific (TNF vs. IL-1beta). Overall, new and interesting findings, which provide further insights into the complex regulation of neutrophil recruitment.

Comments for the author

Rahman and colleagues investigated the role of L-selectin during human neutrophil transendothelial migration (TEM) and identified an interesting new contribution of PECAM-1 and L-selectin (shedding) for proper TEM. Using L-selectin and PECAM-1 (tagged with fluorescent proteins at the C-terminus) expressing THP1 and HL60 cells they show that PECAM-1 and L-selectin co-clustered during TEM (over HUVEC monolayer) following clustering of PECAM-1. Interestingly, the observed co-clustering of L-selectin and PECAM-1 was stimulus dependent. While TNF stimulation induced co-clustering, this was not observed for IL-1beta in both cell lines. The authors then went on and looked at L-selectin shedding which was induced during TEM in TNF-stimulated but not IL-1beta-stimulated neutrophils suggesting that ADAM17 is activated during TEM. Additional experiments then revealed that TEM was much faster when TNF-stimulated (not IL-1beta) HL60 cells were co-transfected with PECAM-1 and L-selectin. Finally, the authors inhibited ADAM17 in primary human neutrophils and found retarded TEM suggesting that blocking L-selectin shedding impairs TEM. This was confirmed in HL60 cells transfected with non-cleavable L-selectin.
Overall, this is an elegant and well-performed study revealing new and interesting insights in the complex regulation of neutrophil recruitment focusing on L-selectin and PECAM-1. I have only a few comments, which should be addressed by the authors:

© 2020. Published by The Company of Biologists under the terms of the Creative Commons Attribution License (https://creativecommons.org/licenses/by/4.0/).

1) The authors transfected cell lines with L-selectin and PECAM-1 (important tool for clustering experimentsl). They also report that WT neutrophils have low expression of those molecules. Interestingly, the TTT is much faster in primary neutrophils than in transfected cells. How do the authors explain this difference also in view of the fact that without transfection TEM is impaired in the cell lines.
2) It is interesting to see that IL-1beta stimulation does not induce L-selectin shedding but still TEM works fine. How do the authors explain this.
3) Figure 2A: TNF: Why do you see two cells with L-selectin staining while only one cell is appearing for CD31 staining?
4) Please include information on the generation of mutant cell lines in the M&M section.

Reviewer 3

Advance summary and potential significance to field

The manuscript by Rahman et al. provides a succinct piece of work describing the contribution of the adhesion receptor L-selectin (CD62-L) to neutrophil transendothelial migration (TEM), a key event that leads to efficient neutrophil recruitment to tissues during inflammation. The principal novelty of the work relies on extending the roles of L-Selectin beyond its well-recognised function in mediating leukocyte tethering and rolling during the initial steps of the leukocyte adhesion cascade. Furthermore, the authors shed light on how the crosstalk between adhesion molecules is necessary for regulating TEM, a phenomenon that has been extensively studied in endothelial cells (ECs) but far less in the myeloid cell compartment. Another strength of the work is that it employs a combination of in vitro approaches to address its hypothesis, including sophisticated imaging techniques to interrogate proximity between adhesion molecules. Although care should be taken when extrapolating in vitro results to in vivo scenarios wherever possible, the authors validate the results obtained with the neutrophil-like HL60 cell line in primary human neutrophils. Specifically, the work elegantly shows how the spatiotemporal clustering of L-Selectin during neutrophil TEM regulates its shedding from the cell surface, a molecular event that accelerates neutrophil TEM. In investigating the potential associated molecular mechanisms, the authors show PECAM-1-mediated co-clustering of L-Selectin is key in mediating the above. The work also provides mechanistic insights into how PECAM-1 modulates this response through its cytoplasmic tail. Intriguingly this mechanism proves to be cytokine-specific, supporting the notion that neutrophil trafficking responses can be governed by the nature of the inflammatory scenario and potentially lead to different immune outcomes.

Overall the work is well conducted, and the experiments are appropriately designed and interpreted. However, I have some major and minor points that should be addressed before the manuscript is accepted for publication in Journal of Cell Science, as well as some suggestions that could help to strengthen the work.

Comments for the author

Major points

- Throughout the work, the authors use CXCR2 expressing HL60 cells as a model to investigate neutrophil TEM responses. However, the efficiency of TEM for this cell line was only validated across HUVEC monolayers stimulated with TNFα. Since the authors argue cytokine specificity for L-Selectin and PECAM-1 co-clustering during TEM, as well as for L-Selecting shedding and neutrophil TEM speed, the efficiency of CXCR2-expressing HL60 cells to fully transmigrate IL-1β stimulated HUVECs should be addressed. The latter will rule out the possibility that, in response to IL-1β, HL60 deficient TEM responses are linked to altered CXCR2 activation instead of PECAM-1 and L-Selectin co-clustering.

- Furthermore, the authors show HL60 cells express low levels of both L-Selectin and PECAM-1 (Supplemental Figure 2). Were the levels of these molecules also addressed in CXCR2-expressing HL60 cells? If so, this should be specified and / or the data provided.

© 2020. Published by The Company of Biologists under the terms of the Creative Commons Attribution License (https://creativecommons.org/licenses/by/4.0/).

• The authors suggest p38MAPK signalling is involved in sensitizing L-Selecting ectodomain shedding induced by PECAM-1 clustering. However, no evidence is provided for p38MAPK activation following PECAM-1 extracellular engagement. Addressing the latter using HL60 cells and / or primary neutrophils would further support the author´s hypothesis.

• Figure 5 indicates PECAM-1 expression in HL60 cells is sufficient to accelerate TEM in response to IL1B, but the administration of HEC7 blocking antibody does not impact HL60 TEM in this context.
Discussing how PECAM-1 expression might be driving neutrophil TEM through IL-1β stimulated HUVECs independently of EC-neutrophil PECAM-1 homophilic interactions will help the reader understand this discrepancy.

• The images in panel E of Figure 7 show HL60 cells expressing the PECAM-1 mutant YYFF exhibit a very different morphology to the control cells expressing WT PECAM-1, indicating cell polarity might be affected during migration in YYFF expressing cells. In order to support YYFF is driving slower TEM due to defective L-Selectin / PECAM-1 co-clustering, the quantification of L-Selecting shedding in these experiments would be very revealing.

• Although the authors show tyrosine residues Y663 and Y686 in PECAM-1 cytoplasmic tail are fundamental for L-selectin co-clustering, the downstream molecular mechanisms leading to L-Selectin clustering are unclear. Since PECAM-1 has been shown to translocate into lipid rafts following its extracellular engagement and the phosphorylation of these tyrosine residues (Florey et al. 2010), does L-Selectin translocate into ordered membrane lipid domains following PECAM-1 mediated clustering? Is PECAM-1 engagement leading to actin remodelling that further leads to L-Selectin clustering? If the authors are not able to answer these questions with experimental data, it would be worth to address these points in the discussion.

Suggestions
• In relation to the above, the authors have previously shown that the spatiotemporal interaction of L-Selectin with the actin-binding ERM proteins governs monocyte protrusion through ECs and L-Selectin ectodomain shedding. The latter is included in the working model represented in Figure 8. Thus, the work would be significantly improved if the authors could address whether ERM proteins are involved in mediating L-Selectin 'inside-out' clustering in response to PECAM-1 engagement. Moreover, are ERM proteins required for L-Selectin shedding following PECAM-1 clustering?

Minor points
• The authors use Manders´Overlap Coefficient to asses association between PECAM-1 and L-Selectin clusters but only provide M2 values. Since both molecules distribute predominantly to the cell surface, M1 and M2 coefficients should offer similar results and providing both parameters will contribute to strengthen the data.
• Figure 1: It would be helpful for the reader to label in the figure panels which experiments were performed in THP-1 cells or primary human monocytes or neutrophils
• Figure S1: The labelling of the black and orange bars represention is mistaken in the figure legend (black bars should correspond to the cells expressing CXCR2).
• Figure 6: The time of TEM should be expressed using the same units in all graphs and figures (either minutes or seconds).
• Figure 7: The data would flow better if all the FRET efficiency results are discussed in the first place and the last two panels refer to the TTT data.

First revision

Author response to reviewers' comments

We are grateful for all of the comments submitted by the reviewers. Below is a point-by- point response to reviewer's comments. For ease of reading, we have added all of our responses in

© 2020. Published by The Company of Biologists under the terms of the Creative Commons Attribution License (https://creativecommons.org/licenses/by/4.0/).

bold italic.

Reviewer 1 Advance Summary and Potential Significance to Field:
The paper describes a role for clustering of L-selectin by PECAM during leukocyte transmigration. The clustering of L-selectin favours shedding and increases the rate of transmigration across TNF-alpha stimulated but not IL-1 stimulated HUVEC monolayers. Shedding deficient L-selectin or mutations in PECAM reduce the rate of TNFalpha dependent but not IL-1 dependent transmigration. This is the first time a role for P-selectin in transmigration has been described.

Reviewer 1 Comments for the Author:
Although an interesting finding the differences between the mechanism of transmigration across IL-1 vs TNF alpha stimulated HUVEC is unclear and this is central to understanding the process; does homotypic PECAM interactions happen with both cytokine stimulation? What is different about the process occurring in IL-1, why is clustering not required in this situation.

We totally agree with Reviewer 1 that this is an important question to address, however, the mechanism that this study focuses on is the novel finding that L-selectin co-clusters with PECAM-1 during TEM, and this has never been witnessed for a cell adhesion molecule that has hitherto been characterised to exclusively mediate tethering and rolling.

We have partially answered the issue of PECAM-1 homotypic interaction using the HEC7 monoclonal antibody, which binds to immunoglobulin (Ig) domains 1 and 2 of PECAM-1 - the very domains that are required for homotypic interactions. In this setting, we have indeed identified that TNF-α-induced TEM requires Ig domains 1 & 2 of PECAM-1 for optimal TTT, whereas TEM across IL-1β-activated HUVEC monolayers is not impacted by blocking Ig domains 1 and 2. This is an important finding, and casts light on the mechanism involved (i.e. Ig domains 1 & 2 of PECAM-1 are used differently to support TEM in a cytokine-dependent manner) - a hitherto unobserved phenomenon. In our view, we have unearthed some molecular mechanism in <u>primary human neutrophils</u>.

There is likely to be a "factor X" that it differentially expressed when endothelial monolayers are stimulated with either TNF-a or IL-1β. Future experiments geared towards understanding the mechanism involved would entail biotinylation of HUVEC monolayers, stimulated with the 2 different cytokines, followed by mass spectrometric analysis of streptavidin-captured proteins. Experiments such as this, as I hope Reviewer 1 would appreciate, falls beyond the scope of this report. We have however elaborated on this X-factor in the discussion section.

We have kept the story linked more towards the novel identification that L-selectin and PECAM-1 co-cluster during TEM, and what the biological significance underpinning this original finding (optimising the TTT through augmenting ectodomain shedding of L-selectin). We think that whilst we are not 100% aware of the mechanism involved, we have uncovered some important highly novel aspects of leukocyte trafficking that will be very important for researchers in the field and bear fruit for future research.

Does this argue that overall clustering isn't that important? How specific is the effect of PECAM clustering given it appears clustering CD43 can have a similar role.

On the contrary, by performing the FRET/FLIM/FRET-based experiments are we able to witness that these interactions are happening within a scale ≤ 10 nm in distance. We show that cell adhesion molecules, such as L-selectin, can cross-talk to other cell adhesion molecules during a specific cellular behaviour, such as TEM. As mentioned in our discussion, these findings highlight that cell adhesion molecules do not have constrained roles within a given stage of the multi-step adhesion cascade.

It is also important to note, as shown in Figure 1A, PSGL-1 and L-selectin have been shown to co-cluster in mice, but not in humans. This has been explained in the original text (along with appropriate references). We have used antibodies to cluster other cell adhesion

© 2020. Published by The Company of Biologists under the terms of the Creative Commons Attribution License (https://creativecommons.org/licenses/by/4.0/).

molecules and did not see co-clustering with L-selectin, suggesting that our observations are specific. We have also used DREG56 (an anti-L-selectin-specific monoclonal antibody), followed by clustering with a secondary antibody as a positive control to determine maximal clustering of L-selectin.

CD43 plays a role in tethering and rolling and we were much more interested in the novel concept of L-selectin co-clustering with a cell adhesion molecule involved in TEM, which is why we focused on this aspect alone. Branching out into how and why L-selectin co-clusters with CD43 would have lost the focus of our work on TEM.

There is no evidence for a difference in neutrophil function following transmigration via a shedding dependent (TNF alpha) or non-dependent (Il-1) route. Does shedding play an important role in governing neutrophil function like it does in monocytes? There is no evidence that this role is important in physiological settings.

Our findings are cell biological, which is why we believe our work falls within the remit of the Journal of Cell Science. This work very much focuses on the highly novel aspect of L-selectin and TEM in neutrophils. As reviewer 3 states – very little has been explored in regard of what regulates neutrophil TEM, so we believe that focusing on TEM more than on the effector functions (in this report) is extremely novel and important.

Recent evidence has shown that blocking shedding of L-selectin affects neutrophil chemotaxis, which could suggest mechanisms beyond TEM would be impacted. We have now added this recent reference from the Zarbock group and have included a section in the discussion, with the following reference:
Cappenberg, A., Margraf, A., Thomas, K., Bardel, B., McCreedy, D. A., Van Marck, V., Mellmann, A., Lowell, C. A. and Zarbock, A. (2019). L-selectin shedding affects bacterial clearance in the lung: a new regulatory pathway for integrin outside-in signaling. Blood 134, 1445-1457.

Overall the statement that L-selectin regulates neutrophil transendothelial migration appears true but only in specific circumstances.

Indeed, the abstract clearly states that the role L-selectin plays in TEM is cytokine-specific. Moreover, the vast majority of reports describing mechanisms of neutrophil TEM across endothelial monolayers is in response to TNF-α rather than IL-1-β.

Comments in detail
Generally the data presented throughout would be better presented if shown as individual data points to get a better idea of data spread and variability.
This has now been changed in the way that Reviewer 1 has suggested.

Fig.1
A-There is a clear difference in L-selectin clustering between cross-linking antibodies, however the fact CD43 can cluster L-selectin to the same magnitude to PECAM surely indicates this is not a specific interaction.

As stated above, we have used 3 other monoclonals where this has not produced a similar co-clustering phenomenon. We therefore believe that this is a specific phenomenon that occurs across via TNF-α-activated endothelial monolayers and not IL-1β-activated monolayers. Moreover, it opens up the importance in receptor cross-talk between different cell adhesion molecules. If anything, this exposes the complexity of how cell adhesion molecules may contribute to unique signals during cell rolling (perhaps in the case of CD43 and L-selectin) and TEM (as in the case of PECAM-1 and L-selectin).

How does the FRET efficiency vary with shape? All the images of cells with high FRET efficiency appear more rounded.

These images were randomly selected by a contributing author (Prof. Maddy Parsons) and

© 2020. Published by The Company of Biologists under the terms of the Creative Commons Attribution License (https://creativecommons.org/licenses/by/4.0/).

cell shape was not a feature of the FLIM/FRET study. The images were captured using a confocal microscope, and some of the optical sections were closer to the coverslip than others. Cells were bound non-specifically on to poly-L-lysine-coated coverslips after clustering. The images were added to represent the fact that FRET can be easily quantified using FLIM (shown in pseudocolour scale), rather than the clustering impacting cell shape on a highly charged substrate (PLL). As shape was not an obvious feature in the TEM assays (subsequent figures), we would not consider this to add value to the TEM story.

B,C and D- There does appear to be greater overlap between PECAM and L-selectin however in some situations the PECAM and L-selectin localisation appears to change to larger vesicular structures see C (PECAM X-link). Given the secondary crosslinker is added at 37C for 25 min, can you rule out co-internalisation? Some of the punctae in D also appear to be inside the cell.

We have provided FACS data (monitoring the levels of L-selectin from at least 10,000 cells) to show that L-selectin levels drop by 22% after clustering with anti-PECAM-1 antibody (HEC7) and secondary antibody, and to about 3% with HEC7 alone. This is now included in Figure 1E and supplementary Figure 1. So, we believe that the small loss in surface expression of L-selectin after clustering PECAM-1 with 1ary plus 2ary antibodies could lead to its internalisation. Whether this phenomenon occurs during TEM and without antibody clustering is currently not known and is elaborated on in the discussion.

Fig.2
This figure shows a convincing difference between IL-1 and TNFalpha treated cells. The figure should also provide evidence that the cells are undergoing TEM (which presumably the authors already have eg. brightfield microscopy).

We are pleased that Reviewer 1 acknowledges that there is a convincing difference in the FRET efficiencies between different cytokine-activated monolayers. TEM is observed as the change in phase contrast imaging from "phase bright" to "phase dark". A movie of this change is represented in movie 3 of the supplementary information, along with Figure 5A and the associated legends. We did not move this in to Figure 2, as the work focuses more on FRET rather than TTT. However, it's important to state that the flow experiments in figures 2 and 3 follow slightly different protocols: FRET measurements in Fig.2 were taken from cells captured in mid-TEM. These images were taken by counterstaining fixed flow assay specimens with anti-PECAM-1 to identify where the endothelial cell junctions have been breached. This is shown in Figure 3 A. We have elaborated on the FRET method in the Materials and Methods section (1st paragraph) to make clearer what had been performed and refer to Figure 3A as an example of where cells have been captured in mid-TEM.
We have also added the following sentence in the legend of Figure 2 to be clear: "Given that endothelial PECAM-1 is enriched at cell-to-cell junctions, FLIM/FRET measurements were acquired in dHL-60 cells captured in mid-TEM (i.e. within regions that were neither exclusively above nor below the endothelium)."

Fig.3
This figure demonstrates there is an effect of blocking L-selectin shedding on levels of L-selectin in TNF alpha but not IL-1. An image of L-selectin levels before transmigration would have helped interpretation of this data as would co- staining with PECAM. Are there any changes in PECAM levels following IL-1 or TNF alpha treatment?

We have now included an image in Fig. 3A to show an adherent neutrophil beside a neutrophil captured in mid-TEM, showing that L-selectin is not only expressed on apically adhered neutrophils but also in the pseudopods of transmigrating neutrophils (white arrowhead). PECAM-1 staining is also used in this figure to show the distribution of PECAM-1 in the transmigrated and non-transmigrated regions of the neutrophil.

Reviewer 1 asks an important question with respect to PECAM-1 expression. We have provided a Western blot in Fig. 3B of the manuscript and quantification data to show that neither IL-1β nor TNF-α affects PECAM-1 expression levels by Western blotting (n=3).

© 2020. Published by The Company of Biologists under the terms of the Creative Commons Attribution License (https://creativecommons.org/licenses/by/4.0/).

Fig.4
This figure provides evidence that PECAM crosslinking can increase PMA mediated shedding in a manner that is dependent on MAPK. This experiment seeks to replicate the clustering and shedding that the authors hypothesise occurs as the cells transmigrate. However the clustering agent, the means to induces shedding and the kinetics of the experiment all differ from what is thought to occur during leukocyte transmigration. So although there is clear experimental differences shown there is no evidence that it mimics anything physiological.

It's extremely difficult to mimic a physiological stimulus for an ectodomain shedding assay, as we trust reviewer 1 can appreciate (particularly if FACS is the readout and cells are subjected to in-suspension assays). The likelihood is that in the flow assay many more receptors are contributing to signals that drive L-selectin shedding, e.g. chemokine receptors and the integrins Mac-1 and LFA-1. However, it is highly likely that classical PKCs (such as PKC□) play an important role in transducing signals downstream of chemokine receptors and integrins.

Taking that data together, we know that L-selectin and PECAM-1 co-cluster during TEM across TNF-a-activated HUVEC and that blocking the ectodomain shedding of L-selectin in either primary neutrophils or dHL-60 cells reduces TTT. None of these observations are witnessed in cells crossing IL-1β-activated HUVEC, which is an interesting and novel find. The aim of this experiment was to simply determine if the clustering of PECAM-1 (which we know happens during TEM) in any way augments the shedding of L-selectin - and we find that it does when we challenge the cells with the PKC-α inducer - PMA.

Like with most in vitro experiments, this is a reductionist approach to what we believe could be played out in the flow assay.

The experiment performed in Fig. 4 was intended to simply demonstrate that clustering of PECAM-1 with primary antibody alone did not augment ectodomain shedding. However, the clustering of HEC7 with secondary antibody was required to augment the shedding response.

PMA is used as a robust agonist of L-selectin shedding, and it is also why we have taken the effort to titrate PMA down to low doses, which has revealed that clustering PECAM-1 dramatically augments ectodomain shedding – well beyond the 22% reduction in L- selectin expression that is now shown in Figure 1E.

In future, we would like to determine (using for example a cremaster muscle model) whether knocking out PECAM-1, L-selectin or both would impede TTT across TNF-α and not IL-1β stimulated cremaster preps. However, such experiments would be beyond the scope of the study.

Fig.5
B and C This figure provides evidence that, in a cell line, expression of PECAM and L-selectin together expedites transmigration following TNF alpha treatment however PECAM alone is sufficient with IL-1 treatment. Blots demonstrating equal level of expression of each of the constructs would strengthen this data.

We apologise for the oversight of not including a section in the materials and methods section regarding the sorting of cell lines. We have now added a section in the materials and methods that all cell lines were sorted for similar expression levels and have included examples of FACS profiles in Fig. S5.

Fig.6
C and D Shedding resistant L-selectin reduces TM time in TNFalpha and to some extent IL-1 treated cells. C the data is incomplete and the cells expressing delta M-N and CD31 should also be treated with TAPI, presumably it should have no effect.
The data laid out in Fig. 6C is a build-up towards the data in Fig. 6D. We did not feel it

© 2020. Published by The Company of Biologists under the terms of the Creative Commons Attribution License (https://creativecommons.org/licenses/by/4.0/).

necessary to also add TAPI-0 to the sheddase-resistant mutants of L-selectin (ΔM-N). Ultimately, the TAPI-0 is best used on primary human neutrophils.

D There is no data for the effect of TAPI treatment or cells expressing L- selectin alone.
The reason for not using TAPI-0 for sheddase-resistant cells is explained above. The perfusion of HL-60 cells expressing L-selectin alone over IL-1β activated endothelial monolayers is shown in Fig. 5C.

C and D would have benefited from western blots to demonstrate similar expression levels of proteins.

As stated above, information on sorting of cells and examples of expression levels are now provided in Fig. S5.

Fig.7
This figure demonstrates that clustering is dependent on the two ITIM residues in the intracellular domain of PECAM in TNF alpha treated cells but not IL-1 treated cells. These residues are necessary for a normal rate of transmigration in TNF alpha but not IL-1. Western blots would improve the figure giving an idea of receptor expression. In figure 5 C PECAM alone was sufficient to increase the rate of transmigration so the lack of an effect on 7D argues that the ITIM residues are unnecessary for IL-1 dependent transmigration.

As mentioned above, Fig. S5 shows FACS analyses of cell lines.

Reviewer 1's conclusion is correct - the intracellular ITIM domain (based on the HL-60 cell work) and extracellular Ig domains 1 & 2 (based on the primary human neutrophil work) are required for optimal TEM across TNF-α-activated endothelial monolayers but not IL-1β-activated monolayers. This is a molecular dissection that will require more research in the future and is commented upon in the Discussion section.

Fig.8
In TNF alpha stimulated cells. Currently the data supporting a homotypic interaction of PECAM driving L-selectin clustering is only supported by the PECAM blocking antibody. There is no evidence from perturbing the other, endothelial side. There is no evidence that clustering of L-selectin is maintained post transmigration this could be addressed with the shedding resistant L-selectin to uncouple the two events.

Indeed, we have learned more about the mechanism of neutrophil TEM crossing TNF-α-activated endothelial monolayers than we have about IL-1□-driven TEM. Therefore, Fig. 8 reflects our understanding of TEM exclusively from the perspective of a TNF-a-stimulated perspective.
This entire work revolves around L-selectin and its contribution to neutrophil TEM and the cis-interaction between L-selectin and PECAM-1 in the context of TEM and the biological significance of this interaction in cis (i.e. expediting TEM through augmenting L-selectin shedding).
We believe that asking questions relating to endothelial-derived PECAM-1 would deflect from the focus of our study. However, this doesn't take away the importance of the contribution that PECAM-1 has in this context.

Reviewer 2 Advance Summary and Potential Significance to Field:
The authors investigated the contribution of L-selectin on transendothelial migration of human neutrophils. They identify PECAM-1 L-selectin co-clustering and L-selectin shedding through ADAM17 as critical processes during TEM. Interestingly, these mechanisms seem to be stimulus specific (TNF vs. IL-1beta). Overall, new and interesting findings, which provide further insights into the complex regulation of neutrophil recruitment.

© 2020. Published by The Company of Biologists under the terms of the Creative Commons Attribution License (https://creativecommons.org/licenses/by/4.0/).

Reviewer 2 Comments for the Author:
Rahman and colleagues investigated the role of L-selectin during human neutrophil transendothelial migration (TEM) and identified an interesting new contribution of PECAM-1 and L-selectin (shedding) for proper TEM. Using L-selectin and PECAM-1 (tagged with fluorescent proteins at the C-terminus) expressing THP1 and HL60 cells they show that PECAM-1 and L-selectin co-clustered during TEM (over HUVEC monolayer) following clustering of PECAM-1. Interestingly, the observed co-clustering of L-selectin and PECAM-1 was stimulus dependent. While TNF stimulation induced co-clustering, this was not observed for IL-1beta in both cell lines. The authors then went on and looked at L-selectin shedding which was induced during TEM in TNF-stimulated but not IL-1beta-stimulated neutrophils suggesting that ADAM17 is activated during TEM. Additional experiments then revealed that TEM was much faster when TNF-stimulated (not IL-1beta) HL60 cells were co- transfected with PECAM-1 and L-selectin. Finally, the authors inhibited ADAM17 in primary human neutrophils and found retarded TEM suggesting that blocking L-selectin shedding impairs TEM. This was confirmed in HL60 cells transfected with non-cleavable L-selectin. Overall, this is an elegant and well-performed study revealing new and interesting insights in the complex regulation of neutrophil recruitment focusing on L-selectin and PECAM-1. I have only a few comments, which should be addressed by the authors:

1) The authors transfected cell lines with L-selectin and PECAM-1 (important tool for clustering experiments). They also report that WT neutrophils have low expression of those molecules. Interestingly, the TTT is much faster in primary neutrophils than in transfected cells.

How do the authors explain this difference also in view of the fact that without transfection TEM is impaired in the cell lines.

We thank Reviewer 2 for their positive comments on the study.

The HL-60 cell line is purely a cellular model within which we can genetically manipulate cells to ask very specific questions about what might be happening in primary human neutrophils, which, in contrast, cannot be genetically manipulated. Clearly there are differences in the migratory capacities of primary neutrophils and HL-60 cells, and this will always be a caveat of using cell lines. Wherever possible, we have attempted to recapitulate experiments (using for example TAPI-0 or HEC7 antibody) to determine if phenotypes are reproduced between HL-60 cells and primary neutrophils. In the majority of cases, we see that this is indeed the case.

We would like to stress that TEM is not impaired in HL-60 cell lines lacking the ectopic expression of L-selectin and PECAM-1. As shown in Figures 5 B&C, the reference cell expressing GFP alone has a TTT of approximately 10 min across TNF-α- or IL-1β-stimulated HUVEC monolayers. What is absolutely crucial to the TEM of primary human neutrophils is the ectopic expression of the chemokine receptor CXCR2. Supplementary fig. 3 shows that the stable expression of CXCR2 is what absolutely drives TEM in these cell lines. We have quoted Jan Lammerding and Ronen Alon's J Leuk Biol paper in the section describing the use of HL-60 cells lines expressing CXCR2 and driving TEM.

2) It is interesting to see that IL-1beta stimulation does not induce L-selectin shedding but still TEM works fine. How do the authors explain this.

This observation fascinates me, as I've been working in the field addressing molecular mechanisms underpinning L-selectin-dependent adhesion and migration for a while now. Currently, we are unaware of what is driving this difference, and the question is likely to be too complicated to tease out in this report. We are hoping in the future to understand this through proteomics and surface biotinylation experiments of endothelial cells treated with the two different cytokines (TNF & IL-1). But, as mentioned above, these sorts of experiments fall outside the remit of this study.

3) Figure 2A: TNF: Why do you see two cells with L-selectin staining while only one cell is appearing for CD31 staining?

On the rare occasion, there are cells that do not express both fluorochromes, and these are

© 2020. Published by The Company of Biologists under the terms of the Creative Commons Attribution License (https://creativecommons.org/licenses/by/4.0/).

obviously excluded from the study.

4) Please include information on the generation of mutant cell lines in the M&M section.

This was an omission on our part, for which we apologise, and has now been included in the Materials and Methods section.

Reviewer 3 Advance Summary and Potential Significance to Field:
The manuscript by Rahman et al. provides a succinct piece of work describing the contribution of the adhesion receptor L-selectin (CD62-L) to neutrophil transendothelial migration (TEM), a key event that leads to efficient neutrophil recruitment to tissues during inflammation. The principal novelty of the work relies on extending the roles of L-Selectin beyond its well-recognised function in mediating leukocyte tethering and rolling during the initial steps of the leukocyte adhesion cascade. Furthermore, the authors shed light on how the crosstalk between adhesion molecules is necessary for regulating TEM, a phenomenon that has been extensively studied in endothelial cells (ECs) but far less in the myeloid cell compartment. Another strength of the work is that it employs a combination of in vitro approaches to address its hypothesis, including sophisticated imaging techniques to interrogate proximity between adhesion molecules. Although care should be taken when extrapolating in vitro results to in vivo scenarios, wherever possible, the authors validate the results obtained with the neutrophil-like HL60 cell line in primary human neutrophils.
Specifically, the work elegantly shows how the spatiotemporal clustering of L-Selectin during neutrophil TEM regulates its shedding from the cell surface, a molecular event that accelerates neutrophil TEM. In investigating the potential associated molecular mechanisms, the authors show PECAM-1-mediated co-clustering of L-Selectin is key in mediating the above. The work also provides mechanistic insights into how PECAM-1 modulates this response through its cytoplasmic tail. Intriguingly, this mechanism proves to be cytokine-specific, supporting the notion that neutrophil trafficking responses can be governed by the nature of the inflammatory scenario and potentially lead to different immune outcomes.
Overall the work is well conducted, and the experiments are appropriately designed and interpreted. However, I have some major and minor points that should be addressed before the manuscript is accepted for publication in Journal of Cell Science, as well as some suggestions that could help to strengthen the work.

Reviewer 3 Comments for the Author: Major points
•Throughout the work, the authors use CXCR2 expressing HL60 cells as a model to investigate neutrophil TEM responses. However, the efficiency of TEM for this cell line was only validated across HUVEC monolayers stimulated with TNFα. Since the authors argue cytokine specificity for L-Selectin and PECAM-1 co-clustering during TEM, as well as for L-Selecting shedding and neutrophil TEM speed, the efficiency of CXCR2-expressing HL60 cells to fully transmigrate IL-1β stimulated HUVECs should be addressed. The latter will rule out the possibility that, in response to IL-1β, HL60 deficient TEM responses are linked to altered CXCR2 activation instead of PECAM-1 and L-Selectin co-clustering.

The data presented in Fig. S3 (previously Fig. S2) was a proof of concept experiment to demonstrate that ectopic expression of CXCR2 is absolutely essential for dHL-60 TEM. The use of TNF-a in these experiments was purely part of the proof-of-concept. This work (unfortunately for our group) has since been validated by another group (as cited in the manuscript - Yadav et al 2018).
The green data points in Fig. 5B and C represent the TEM times of dHL-60 cells expressing GFP alone (i.e. no ectopic expression of either L-selectin or PECAM-1) crossing either TNF-α or IL-1β-activated HUVEC monolayers. Note: all cells in these experiments are stably expressing CXCR2. We believe that the GFP alone data is more appropriate than the proof of concept data in Fig. S2, as the HL-60 cells stably expressing GFP have been infected with lentivirus - controlling for the lentiviral infection of PECAM-1-GFP/L-selectin-RFP-expressing cells.

•Furthermore, the authors show HL60 cells express low levels of both L-Selectin and PECAM-1

© 2020. Published by The Company of Biologists under the terms of the Creative Commons Attribution License (https://creativecommons.org/licenses/by/4.0/).

(Supplemental Figure 2). Were the levels of these molecules also addressed in CXCR2- expressing HL60 cells? If so, this should be specified and / or the data provided.
We apologise for this oversight in explanation. All of the lysates in Fig. S4 (originally Fig. S2) were extracted from HL-60 cells stably expressing CXCR2. The legend to this figure has now been amended.

•The authors suggest p38MAPK signalling is involved in sensitizing L-Selecting ectodomain shedding induced by PECAM-1 clustering. However, no evidence is provided for p38MAPK activation following PECAM-1 extracellular engagement. Addressing the latter using HL60 cells and / or primary neutrophils would further support the author´s hypothesis.

We have now included data to show that clustering endogenous PECAM-1 in primary human neutrophils activates a number of different MAPKs, including p38MAPKδ (see Figure 4A). The MAPK array presented in Figure 4A has been performed across 2 independent experiments, so we are confident that this result is reproducible. We also show that JNK and Akt are also robustly activated. Importantly, this data validates the contribution of PECAM-1 clustering to the activation of p38MAPK and its involvement in sensitising L-selectin for ectodomain shedding.
In figure 4D, we show that inhibiting JNK in primary neutrophils paradoxically promotes L-selectin shedding. We could therefore not subject neutrophils to the same PMA dose-dependent shedding assays as with the p38MAPK inhibitor. This outcome now leads us to hypothesise that activation of JNK by PECAM-1 clustering may protect L-selectin from being cleaved - possibly in the early stages of TEM, where we hypothesise that L-selectin plays a role in signalling to drive pseudopod protrusion. We have added some comments in the Discussion section on how, upon PECAM-1 clustering, p38MAPKδ, activation is sustained, but in contrast JNK activation is only transiently switched on to protect L-selectin from shedding during TEM.

•Figure 5 indicates PECAM-1 expression in HL60 cells is sufficient to accelerate TEM in response to IL1B, but the administration of HEC7 blocking antibody does not impact HL60 TEM in this context. Discussing how PECAM-1 expression might be driving neutrophil TEM through IL-1β stimulated HUVECs independently of EC-neutrophil PECAM-1 homophilic interactions will help the reader understand this discrepancy.

We have added a section in the discussion that will elaborate on the impact of HEC7 in blocking neutrophil-derived PECAM-1 from interacting with HUVEC-derived PECAM-1, and how this axis of interaction appears to be more important for TEM across TNF-α-activated endothelial monolayers, but not IL-1β-activated monolayers. We have included a section in the discussion that suggests an "X-factor" that could be up-regulated under IL-1b and not TNF-a activation, and how this factor may overcome the need for ITIM and the Ig domains 1&2.

•The images in panel E of Figure 7 show HL60 cells expressing the PECAM-1 mutant YYFF exhibit a very different morphology to the control cells expressing WT PECAM-1, indicating cell polarity might be affected during migration in YYFF expressing cells. In order to support YYFF is driving slower TEM due to defective L-Selectin / PECAM-1 co-clustering, the quantification of L-Selecting shedding in these experiments would be very revealing.

Whilst the images for FRET may indicate changes in cellular morphology, we did not see any obvious differences in the live cell imaging during the flow assays. Labelling cells with LifeAct to monitor the overall cellular morphologies would be interesting to determine in future experiments. We have made a comment in the Discussion section that this would be an interesting future experiment to conduct, along with other cell lines expressing the ERM-binding mutant of L-selectin.

•Although the authors show tyrosine residues Y663 and Y686 in PECAM-1 cytoplasmic tail are fundamental for L-selectin co-clustering, the downstream molecular mechanisms leading to L-Selectin clustering are unclear. Since PECAM-1 has been shown to translocate into lipid rafts following its extracellular engagement and the phosphorylation of these tyrosine residues

© 2020. Published by The Company of Biologists under the terms of the Creative Commons Attribution License (https://creativecommons.org/licenses/by/4.0/).

(Florey et al. 2010), does L-Selectin translocate into ordered membrane lipid domains following PECAM-1 mediated clustering? Is PECAM-1 engagement leading to actin remodelling that further leads to L-Selectin clustering? If the authors are not able to answer these questions with experimental data, it would be worth to address these points in the discussion.

We agree with Reviewer 3 that this would be a useful aspect to elaborate, so we have included more information in Figure 8 and the associated legend.

Suggestions
•In relation to the above, the authors have previously shown that the spatiotemporal interaction of L-Selectin with the actin-binding ERM proteins governs monocyte protrusion through ECs and L-Selectin ectodomain shedding. The latter is included in the working model represented in Figure 8. Thus, the work would be significantly improved if the authors could address whether ERM proteins are involved in mediating L-Selectin 'inside- out' clustering in response to PECAM-1 engagement. Moreover, are ERM proteins required for L-Selectin shedding following PECAM-1 clustering?

This is definitely a great suggestion made by reviewer 3 and we have made a comment on this in the discussion section. Our future work will aim to look at these aspects in greater detail.

Minor points
•The authors use Manders´Overlap Coefficient to asses association between PECAM-1 and L-Selectin clusters but only provide M2 values. Since both molecules distribute predominantly to the cell surface, M1 and M2 coefficients should offer similar results and providing both parameters will contribute to strengthen the data.

Our response to Reviewer 3's comment is a bit long-winded, but we hope that we have put our point across to explain why we have only used M2 in our study:

Manders Colocalisation Coefficients, M1 and M2, provide the fraction of overlap between signals emanating from two independent fluorescence probes. Each coefficient defines the fraction of signal from one probe in a compartment that already contains signal from the other probe[1]. Within the confines of this study, the aim is to focus on how efficiently antibody clustering of one molecule i.e. PECAM-1, draws the other "untouched" molecule i.e. L-selectin, to localize in close proximity. Based on commands set in the Volocity software, this directional effect is characterized by M2 (fraction of clustered signal overlapped by signal of the untouched molecule), and why we have used only M2 in the data. M1 in this scenario would simply characterize the reverse, which is how effectively the antibody is corralling one molecule towards the other.

In terms of whether the coefficients would be similar, we believe that this would not be the case, especially in the cell lines where GFP-tagged L-selectin constructs are expressed. Whilst M2 accounts for the fraction of the PECAM-1 clustered signal that contains signal of GFP-tagged L-selectin, M1 would describe the reverse. The PECAM-1 clustering occurs at the plasma membrane (e.g. during TEM in the "real" scenario) so the translocation of L-selectin to these areas of clustered signal is measured through M2. The M1 value would give the fraction of total "untouched" L-selectin-GFP (including the cytosolic signal) that is overlapped by membrane PECAM-1 signal, hence the M1 value will be significantly lower. Analysis of colocalisation in this direction is not a sufficient way to understand cross-talk that is driven by PECAM-1 clustering. Therefore, it highlights the importance of placing more focus on one coefficient over the other depending on what is being investigated. The difference between the two coefficients determines how important it is to focus in on one of the two coefficients - therefore underlining the value of separating out M1/M2 in understanding colocalisation. In fact, this is demonstrated aptly by Manders et al in their study published in 1993. They show in Table 1 how M1 & M2 can differ significantly when colocalisation is analysed, where signals from two distinct probes/sources have been overlaid into the same image[2].

1 Dunn KW, Kamocka MM, McDonald JH. A practical guide to evaluating colocalization in

© 2020. Published by The Company of Biologists under the terms of the Creative Commons Attribution License (https://creativecommons.org/licenses/by/4.0/).

biological microscopy. Am J Physiol Cell Physiol. 2011;300(4):C723-C742
2 Manders, E.M.M., Verbeek, F.J. and Aten, J.A. (1993), Measurement of co-localization of objects in dual-colour confocal images. Journal of Microscopy, 169: 375-382

•Figure 1: It would be helpful for the reader to label in the figure panels which experiments were performed in THP-1 cells or primary human monocytes or neutrophils

This has now been amended.

•Figure S1: The labelling of the black and orange bars represention is mistaken in the figure legend (black bars should correspond to the cells expressing CXCR2).
We apologise for this oversight and thank Reviewer 3 for their keen eye in spotting this! Fig. S1 is now Fig. S3.

•Figure 6: The time of TEM should be expressed using the same units in all graphs and figures (either minutes or seconds).

This has now been adjusted.

•Figure 7: The data would flow better if all the FRET efficiency results are discussed in the first place and the last two panels refer to the TTT data.

We thank Reviewer 3 for this suggestion. The amendment has now been made.

Second decision letter

MS ID#: JOCES/2020/250340

MS TITLE: L-selectin regulates human neutrophil transendothelial migration

AUTHORS: Izajur Rahman, Aida Collado Sanchez, Jess Davies, Karolina Rzeniewicz, Sarah Abukscem, Justin Joachim, Hannah Louise Green, Maria Jesus Sanz, David James Killock, Guillaume Charras, Maddy Parsons, and Aleksandar Ivetic
ARTICLE TYPE: Research Article

I am happy to tell you that your manuscript has been accepted for publication in Journal of Cell Science, pending standard ethics checks.

Reviewer 1

Advance summary and potential significance to field

The paper by Rahman et al uses tagged L-selectin constructs alongside FLIM to detail a role for co-clustering of L-selectin and CD31 during neutrophil transmigration across stimulated endothelium. The clustering of L-selectin when crossing TNF alpha stimulated endothelial cells results in a significant change in shedding and the rate of transmigration. PMA induced shedding can be reduced by inhibition of p38 MAPK. Clustering of CD31 increases the rate of TEM across TNF alpha stimulated, this is dependent on ITIM residues present in CD31Â’s cytoplasmic tail. Throughout this work the authors note a difference between IL-1 and TNF alpha stimulated endothelium. The molecular mechanism for this difference is as yet unclear as is the relevance of this process in a physiological context.

© 2020. Published by The Company of Biologists under the terms of the Creative Commons Attribution License (https://creativecommons.org/licenses/by/4.0/).

Comments for the author

The authors have improved the paper. The clarifications in the methods and the FACs analysis (S5) showing the expression levels of the stable HL60 cells strengthen the conclusions that can be drawn from this aspect of the work.

The additional FACS data in fig S1 demonstrates that the effect of cross linking on the rate of endocytosis is relatively minor.

Plotting the individual data points in the graphs provides a better idea of the spread of the data and the trends that have been noted.

Importantly the authors also included additional speculation as to the role of additional factors in IL-1 stimulation of endothelial cells.

There is still some question as to how relevant the work will be in a more physiological setting (is a delay of 1 to 2 min likely to affect an inflammatory event?). However, overall the publication is suitable for acceptance.

Reviewer 2

Advance summary and potential significance to field

The authors have identified a role of L-selectin during transendothelial migration (TEM) of neutrophils and observed co-clustering of L-selectin with PECAM-1. Co-clustering was associated with an increase in L-selectin shedding which significantly accelerated TEM. Interestingly, this process was stimulus dependent and present in TNF-a stimulated EC, but not in IL-1b stimulated EC.

Comments for the author

The authors have adequately addressed all my concerns.

Reviewer 3

Advance summary and potential significance to field

The manuscript by Rahman et al. describes an unidentified role for L-selectin during neutrophil transendothelial migration. Specifically, the work elegantly shows how the spatiotemporal clustering of L-Selectin during neutrophil TEM regulates its shedding from the cell surface, a molecular event that accelerates neutrophil TEM. In investigating the potential associated molecular mechanisms, the authors show PECAM-1-mediated co-clustering of L-Selectin is key in mediating the above. The work also provides mechanistic insights into how PECAM-1 modulates this response through its cytoplasmic tail and downstream signaling. Intriguingly, this mechanism proves to be cytokine-specific, supporting the notion that neutrophil trafficking responses can be governed by the nature of the inflammatory scenario and potentially lead to different immune outcomes. Overall, the work has been strengthened after revision and provides robust insights into the complex regulation of neutrophil transendothelial migration.

Comments for the author

The authors have addressed all my concerns and I recommend the manuscript for publication in JCS.

© 2020. Published by The Company of Biologists under the terms of the Creative Commons Attribution License (https://creativecommons.org/licenses/by/4.0/).
